# Genetic diversity analysis in a mini core collection of Damask rose (*Rosa damascena* Mill.) germplasm from Iran using URP and SCoT markers

**DOI:** 10.1186/s43141-021-00247-7

**Published:** 2021-09-30

**Authors:** Atefeh Sadat Mostafavi, Mansour Omidi, Reza Azizinezhad, Alireza Etminan, Hassanali Naghdi Badi

**Affiliations:** 1 Department of Plant Breeding and Biotechnology, Science and Research Branch, Islamic Azad University, Tehran, Iran; 2 Department of Agronomy and Plant Breeding, Agricultural College, University of Tehran, Karaj, Iran; 3 Department of Plant breeding and Biotechnology, Kermanshah Branch, Islamic Azad University, Kermanshah, Iran; 4 Medicinal Plants Research Center, Institute of Medicinal Plants, ACECR, Karaj, Iran

**Keywords:** *Rosa damascena* Mill, SCoT, URP, Genetic variation, Population structure

## Abstract

**Background:**

*Rosa damascena* Mill is a well-known species of the rose family. It is famous for its essential oil content. The aim of the present study was to assess the genetic diversity and population structure of a mini core collection of the Iranian Damask rose germplasm. This involved the use of universal rice primers (URP) and start codon targeted (SCoT) molecular markers.

**Results:**

Fourteen URP and twelve SCoT primers amplified 268 and 216 loci, with an average of 19.21 and 18.18 polymorphic fragments per primer, respectively. The polymorphic information content for URR and SCoT primers ranged from 0.38 to 0.48 and 0.11 to 0.45, with the resolving power ranging from 8.75 to 13.05 and 9.9 to 14.59, respectively. Clustering was based on neighbor-joining (NJ). The mini core collection contained 40 accessions and was divided into three distinct clusters, centered on both markers and on the combination of data.

**Conclusion:**

Cluster analysis and principal coordinate analysis were consistent with genetic relationships derived by STRUCTURE analysis. The findings showed that patterns of grouping did not correlate with geographical origin. Both molecular markers demonstrated that the accessions were not genetically diverse as expected, thereby highlighting the possibility that gene flow occurred between populations.

**Supplementary Information:**

The online version contains supplementary material available at 10.1186/s43141-021-00247-7.

## Background

As a large genus in the Rosaceae family, *Rosa* has 200 species and covers more than 18,000 cultivars [1]. The Caucasus, Syria, Morocco, and Andalusia are all home to *Rosa damascena*, while Iran is usually referred to as a source of diversity in this respect [2]. Accordingly, a great variation of Damask rose landraces is expected to be discovered in this country. In addition to horticultural uses, roses are of economic importance because of the essential oils in their petals [3]. *Rosa damascena* has particular genotypes and cultivars which are noteworthy for their medicinal properties and oil [4–6]. Since genetic variation is available within the genus of Rosa, its breeding is usefully dependent on the systematic characterization of genetic resources and the study of likely mechanisms for hybridization. Morphological markers describe an organism’s phenotypic characteristics and are the first to outline an organism’s measurable characteristics. Each species in the Rosa genus has a wide, overlapping range of morphological variations that are affected by environmental factors. Thus, it would be insufficient to classify species and genotypes on morphological ground only [7]. According to Kiani et al. [8], the most of Iranian Damask roses are tetraploid; however, some other ploidy levels were observed.

For the classification and recognition of rose genotypes, chemotaxonomic studies are often addressed in a large variety of different phenolic structures and isozyme markers [8–11]. Nonetheless, there is a limited number of regularly resolvable loci, but this can reduce the efficiency of these markers [9, 12]. The molecular approach is more acceptable because it provides easy access to the genetic material (genome) which makes it much easier to recognize plant relationships [13]. Molecular markers can identify genetic polymorphism at the DNA level and can be used in analyzing genetic variation, genetic distance estimate, parentage determination, marker-assisted selection, and gene localization. Many DNA-based molecular markers are available for the purpose of distinguishing biodiversity among plant populations. However, the selection of DNA markers depends on the type of study. Therefore, it is important to compare the various molecular markers and decide which molecular marker is appropriate for the species under study. New innovations have given rise to new molecular markers that can be used in describing genetic characteristics of plants in the *Rosa* genus. Several molecular assays have been used in recent years to test the genetic variation of various rose plants [14–22]. In theory, these molecular approaches, operations, classes, polymorphic count, function, and time requirements are varied.

In the plant genome, the SCoT marker mechanism relies on the start codon (ATG) which has a short conservation around it [23]. These markers can reproduce well within annealing temperatures [24] and have great potential as a relatively popular tool. SCoT marker system is a simple, low cost, polymorphic, reproducible, and reliable marker system. SCoT markers are known to be useful in a various studies, such as, cultivar recognition, genetic diversity evaluation, DNA fingerprinting, marker assistant selection and quantitative trait loci mapping [25, 26]. This approach has an important context within genetic studies while its benefits are numerous [27–30].

Kang et al. [31] used a polymerase chain reaction (PCR) method using universal rice primers (URP) that provide a powerful tool for investigating the DNA diversity of most eukaryotic and prokaryotic genomes, with potential use in taxonomic and phylogenic research, as well as in population genotypic screening of individuals, both at the inter- and intraspecies level. As a matter of long primers and elevated annealing temperatures, URP-PCR has an advantage over randomly amplified polymorphic DNA (RAPD) and arbitrarily primed polymerase chain reaction (AP-PCR) methods. DNA marker performance is evaluated through factors like the marker index (*MI*) and the polymorphism information content (*PIC*). Comparing the ability of marker techniques can assist researchers in selecting the required markers in the amplification of genome fragments, thereby being more effective in using these markers for potential breeding studies [32].

This study aimed to investigate genetic variation in different *Rosa damascena* accessions from Iran and to demonstrate the effectiveness of different marker systems.

## Methods

### Plant materials and DNA extraction

**Fig. 1:**
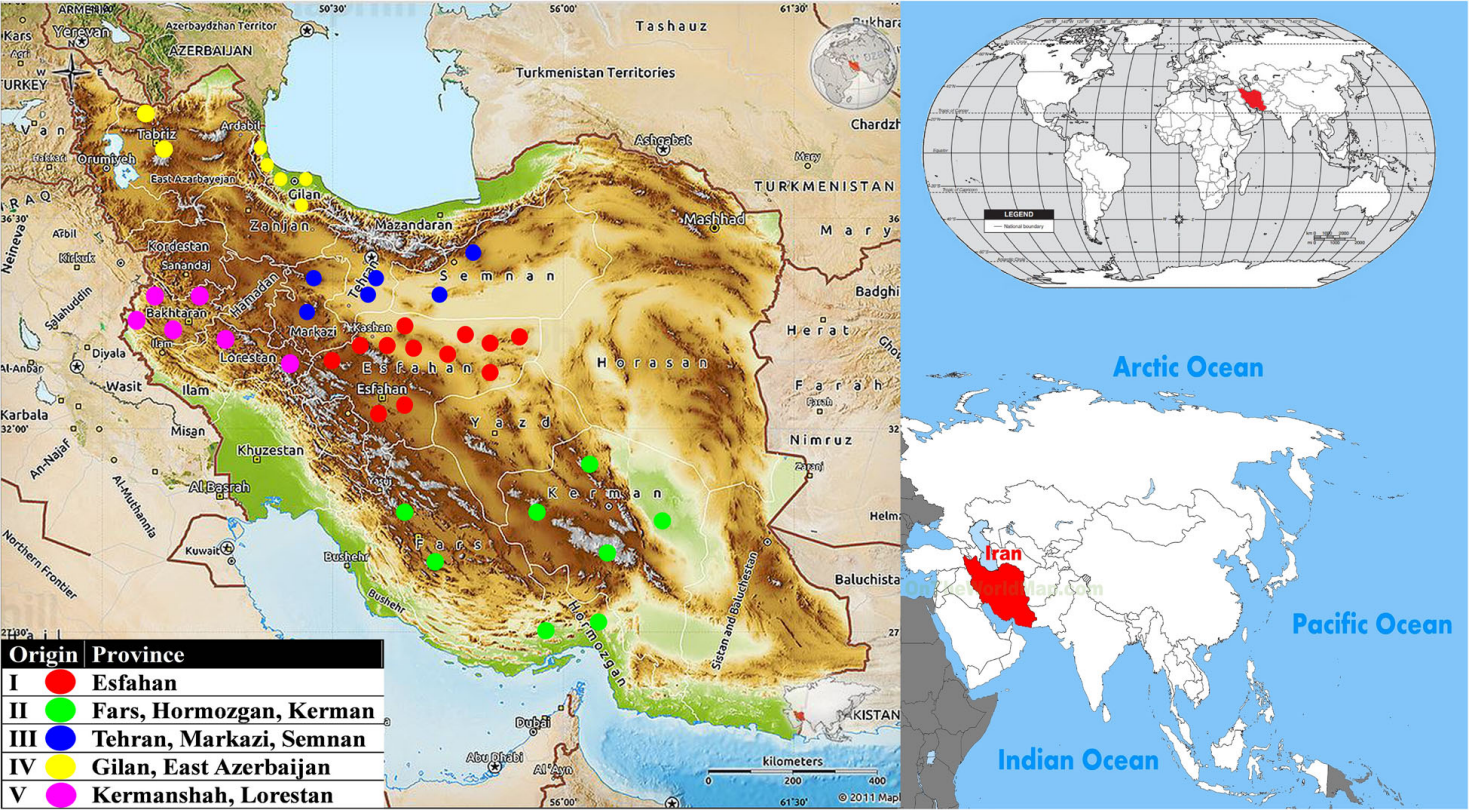
The map shows five preferred regions and their climates from which Iranian damask roses were collected

In total, 40 Damask rose genotypes were collected from five regions in Iran (Fig. 1, Table 1). Sucker roses were harvested from Iran’s rose oil–producing regions. These areas were divided according to geographical and climatological conditions, and each region consisted of some provinces (Fars, Isfahan, East Azerbaijan, Kerman, Semnan, Gilan, Kermanshah, Lorestan, Hormozgan, Tehran, and Markazi provinces). The geographical details are mentioned in Table 1. Accessions have been collected from the gene bank collection of Barij Essence company in Kashan. Young leaves from each accession were collected for DNA extraction since late March to early June. The CTAB procedure [33] was used, with slight modifications (changing the amount and content of the extraction buffer, the incubation time, and adding polyethylene glycol), to extract total genomic DNA. Electrophoresis was performed on a 1% agarose gel to evaluate the quality of DNA. High-quality genomic DNA samples were considered to be without broken DNA for amplification.

**Table 1:** Details of rose accessions used in the study

No.	Accession name	Province	Origin	Longitude (E)	Latitude (N)	Altitude (m a.s.l)	Climate
1	Ghamsar1	Esfahan	Region I	51° 24' 57E	33° 44' 38N	1897	Arid-temperate
2	Ardehal	Esfahan	Region I	51° 3' 1E	34° 2' 18N	1788	Arid-warm
3	Kamu1	Esfahan	Region I	51° 15' 56E	33° 37' 30N	2200	Arid-temperate
4	Azeran	Esfahan	Region I	51° 7' 52E	33° 42' 43N	2334	Arid-temperate
5	Ozvar	Esfahan	Region I	51° 9' 12E	33° 48' 59N	2044	Arid-temperate
6	Ghamsar2	Esfahan	Region I	51° 24' 57E	33° 44' 38N	1897	Arid-temperate
7	Nabar	Esfahan	Region I	51° 12' 12E	33° 52' 18N	1613	Arid-warm
8	Ghohrud	Esfahan	Region I	51° 24' 46E	33° 40' 23N	2300	Arid-warm
9	Barzok1	Esfahan	Region I	51° 13' 38E	33° 47' 9N	2044	Arid-temperate
10	Kamu2	Esfahan	Region I	51° 15' 56E	33° 37' 30N	2200	Arid-temperate
11	Barzok2	Esfahan	Region I	51° 13' 38E	33° 47' 9N	2044	Arid-temperate
12	Barzok3	Esfahan	Region I	51° 13' 38E	33° 47' 9N	2044	Arid-temperate
13	Darab	Fars	Region II	54° 32' 40E	28° 45' 7N	1139	Semiarid-temperate
14	Meymand	Fars	Region II	52° 45' 12E	28° 52' 4N	1532	Arid-warm
15	Shiraz	Fars	Region II	52° 31' 52E	29° 36' 37N	1532	Semiarid-warm
16	Minab	Hormozgan	Region II	57° 5' 14E	27° 7' 52N	27	Humid-warm
17	Hormozgan	Hormozgan	Region II	56° 16' 51E	27° 11' 11N	0	Humid-warm
18	Bardsir	Kerman	Region II	56° 34' 27E	29° 55' 22N	2047	Arid-warm
19	Lalezar	Kerman	Region III	55° 8' 50E	30° 6' 24N	1853	Arid-warm
20	kerman	Kerman	Region III	57° 4' 44E	30° 16' 60N	1788	Arid-warm
21	Sirach	Kerman	Region III	57° 32' 38E	30° 12' 17N	1788	Arid-warm
22	Firuzkuh	Tehran	Region III	52° 46' 14E	35° 45' 24N	1906	Humid-temperate
23	Lavasanat	Tehran	Region III	51° 46' 52E	35° 49' 27N	2556	Semiarid-temperate
24	Ghalhar	Markazi	Region III	50° 59' 56E	33° 53' 34N	2300	Arid-temperate
25	Delijan	Markazi	Region III	50° 41' 2E	33° 59' 26N	1541	Arid-temperate
26	Semnan1	Semnan	Region III	53° 23' 31E	35° 34' 37N	1140	Semiarid-temperate
27	Semnan2	Semnan	Region III	53° 23' 31E	35° 34' 37N	1140	Semiarid-temperate
28	lahijan	Gilan	Region IV	50° 00' 14E	37° 12' 26N	2	Humid-temprate
29	Rasht	Gilan	Region IV	49° 35' 19E	37° 16' 34N	1	Humid-temprate
30	Chaboksar	Gilan	Region IV	50° 35′ 8E	36° 57' 33N	216	Humid-temprate
31	Astaneh	Gilan	Region IV	49° 56' 36E	37° 15' 48N	-2	Humid-temprate
32	Somesara	Gilan	Region IV	49° 18' 54E	37° 18' 5N	0	Humid-temprate
33	Tabriz1	East Azerbijan	Region IV	46° 17' 31E	38° 4' 48N	1394	Humid-cool
34	Tabriz2	East Azerbijan	Region IV	46° 17' 31E	38° 4' 48N	1394	Humid-cool
35	Gareban1	Kermanshah	Region V	47° 23' 33E	34° 9' 8N	1276	Semiarid-cool
36	Gareban2	Kermanshah	Region V	47° 23' 33E	34° 9' 8N	1276	Semiarid-cool
37	Koohdasht1	Kermanshah	Region V	47° 36' 36E	33° 31' 60N	1276	Semiarid-cool
38	Koohdasht2	Kermanshah	Region V	47° 36' 36E	33° 31' 60N	1276	Semiarid-cool
39	Khoramabad	Lorestan	Region V	48° 21' 21E	33° 29' 16N	1347	Semiarid-cool
40	Borujerd	Lorestan	Region V	48° 40' 13E	33° 10' 12N	1276	Semiarid-temperate

### PCR amplification of different markers

The sequence and annealing temperature of all primers for the analysis are given in Table 2. The genomic DNA of all 40 genotypes was amplified with a set of 12 SCoT primers and 14 URP primer sequences [34]. The amplification was done in a Bio-Rad (T100) thermal cycler. Twenty microliters of PCR reaction mixtures consisted of 6.5 μl ddH_2_O, 10 μl master mix 2XPCR (ready-to-use PCR master mix 2X; Ampliqon), 2 μl isolated DNA per sample (50 ng/μl), and 1.5 μl per primer (10 pmole/ml). Each PCR cycle ran on initial denaturating at 94 °C for 5 min, 35 denaturation cycles at 94 °C for 45 s, with a primer annealing time of 45 s (Table 2). This procedure was applied for each primer. Primer elongation lasted for 90 s at 72 °C. A final extension cycle ran for 10 min (72 °C). In order to detect polymorphism among accessions, the PCR product was transferred to 1.2% agarose gel wells, and then electrophoresis was performed at 90 volts. The gel was then immersed in ethidium bromide solution for 15 min (10 mg/ml). Using the gel documentation method, the illustration of banding patterns was obtained under UV light (Bio-Rad). SCoT and URP primers were used on the gel for the amplified fragments.

**Table 2:** URP and SCoT primers and their amplification results generated in the *Rosa damascena* Mill. germplasm

Marker	Primer	Sequence (5 → 3)	*Ta* (°C)	*TAB*	*NPB*	*PPB*	*PIC*	*RP*	*MI*
URP	URP-1	ATCCAGGTCCGAGACAACC	48	20	20	100	0.48	16.35	9.6
	URP-2	CCCAGCAACTGATCGCACAC	48	20	20	100	0.43	13.7	8.6
	URP-3	AGGACTCGATAACAGGCTCC	48	20	20	100	0.44	14.1	8.8
	URP-4	ATGTGTGCGATCAGTTGCTG	48	16	16	100	0.38	8.75	6.08
	URP-6	GGACAAGAAGAGGATGTGGA	48	19	19	100	0.47	14.65	8.93
	URP-8	CCTCCTCCCTCCT	48	20	20	100	0.39	11.65	7.8
	URP-9	AGGGCTGGAGGAGGGC	48	20	20	100	0.38	11.6	7.6
	URP-10	CCTGTGTGTGTGCAT	48	20	20	100	0.43	13.5	8.6
	URP-11	ATGCACACACACAGG	48	18	18	100	0.40	11.85	7.2
	URP-12	GGTGAAGCACAGGTG	48	18	18	100	0.44	12.5	7.92
	URP-13	GGTGTAGAGAGGGGT	48	19	19	100	0.45	13.6	8.55
	URP-15	GGCAGGATTGAAGC	48	20	20	100	0.43	14.35	8.6
	URP-17	AGGAGGAGGGGAAGG	48	20	20	100	0.45	14.5	9
	URP-18	GAGGGTGGCGGCTCT	48	19	19	100	0.40	11.65	7.6
	Mean			19.21	19.21	100	0.42	13.05	8.20
SCoT	SCoT-2	CAA CAA TGG CTA CCA CCC	56	17	17	100	0.38	9.9	6.46
	SCoT-3	CAA CAA TGG CTA CCA C CG	56	17	17	100	0.43	12.05	7.31
	SCoT-4	CAA CAA TGG CTA CCA CCT	54	20	20	100	0.45	14.59	9
	SCoT-5	CAA CAA TGG CTA CCA CGA	54	16	16	100	0.40	10.29	6.4
	SCoT-8	CAA CAA TGG CTA CCA CGT	54	20	20	100	0.38	11.85	7.6
	SCoT-9	CAA CAA TGG CTA CCA GCA	54	18	18	100	0.39	10.63	7.02
	SCoT-11	AAG CAA TGG CTA CCA CCA	54	18	18	100	0.41	11.61	7.38
	SCoT-12	ACG ACA TGG CGA CCA ACG	58	20	20	100	0.45	13.37	9
	SCoT-14	ACG ACA TGG CGA CCA CCG	61	17	17	100	0.43	12.15	7.31
	SCoT-15	ACG ACA TGG CGA CCG CGA	61	18	18	100	0.43	13.51	7.74
	SCoT-21	CAC CAT GGC TAC CAC CAT	56	19	19	100	0.1	13.12	1.9
	SCoT-26	ACA ATG GCT ACC ACC ATC	54	16	16	100	0.44	11.02	7.04
	Mean			18.18	18.18	100	0.37	12.09	7.35

*Ta* temperature annealing, *TAB* total amplified bands, *NPB* number of polymorphic, *PPB* percentage of polymorphism, *PIC* polymorphism information content, *RP* resolving power, *MI* marker index

### Data analysis

The amplified fragments were scored as absent (0) or present (1) in each sample. Screening the primers involved using several discriminatory criteria, including the number of polymorphic bands (*NPB*), total amplified bands (*TAB*), percentage of polymorphism bands (*PPB*), resolving power (*Rp*), polymorphism information content (*PIC*), and marker index (*MI*). *PIC* was calculated based on the formula given by Anderson et al. [35].

Molecular variation analysis (AMOVA) operated via GenAlEx ver. 6.5 to classify genetic diversity [36]. For each sample, GenAlEx ver. 6.5 was used for determining the percentage of polymorphic loci (*PPL*), effective number of alleles (*Ne*), and total number of alleles (*Na*) [37], Nei’s [38] gene diversity (*H*), and Shannon’s information index (*I*) [39]. Then, Jaccard’s method was used for finding genetic dissimilarities by DARwin ver. 6 software [40]. The neighbor-joining (NJ) method contributed to the construction of the Fan-dendrogram using MEGA ver. 10.1 software [41]. The genetic makeup of populations was analyzed by the Bayesian-based model. This was performed by STRUCTURE (ver. 2.3.4) [42]. It estimated the clusters of population genetics (*K*) and the ratio of individual assignment out of each population. For each ‘*K*’ varying from 1 to 10, the analysis was repeated ten times, and the initial burn-in period was set to 100,000 followed by 100,000 Markov Chain Monte Carlo (MCMC) iterations. Finally, the DK was calculated by STRUCTURE HARVESTER, an online program [42].

## Results

### URP and SCoT polymorphism

In this analysis, the genetic polymorphism of *Rosa damascena* was tested using 14 URP and 12 SCoT primers. Table 2 gives a description of the informativeness criteria being calculated for URP and SCoT primers. There were 268 amplified fragments among the 40 accessions. These were amplified by all URP primers and turned out to be entirely polymorphic. The polymorphic band count was from 16 (URP-4) to 20 (URP-1, URP-2, URP-3, URP-8, URP-9, URP-10, URP-15, and URP-17), while averaging at 19.21. There was a variation in PIC values from 0.38 (URP-4) to 0.48 (URP-1) with an average of 0.42. The average value of resolving power (Rp) was 13.05 and URP-1 which displayed the highest value (16.35), although its lowest (8.75) belonged to URP-4. The highest value of MI was measured for URP-1 (9.6), while the lowest value was associated with URP-4 (6.08). In SCoT, 12 primers generated 216 loci, all being polymorphic fragments. The total bands per primer varied between 16 (SCoT-5, SCoT-26) and 20 (SCoT-4, SCoT-8, SCoT-12). The PIC values were between 0.11 (SCoT-21) and 0.45 (SCoT-4, SCoT-12), while having an average of 0.37. The Rp value ranged from 9.9 (SCoT-2) to 14.59 (SCoT-4) for the twelve primers, thereby distinguishing between various genotypes. The lowest and highest values of MI occurred in SCoT-21 (1.9), SCoT-12 and SCoT-4 (9), respectively.

### Genetic diversity analysis

Variations among and within the *Rosa damascena* populations were detected by molecular variance analysis (AMOVA) (Table 3). The results of AMOVA showed a higher molecular variation (%) within populations (URP = 96%, SCoT = 90%, combined data = 93%), compared to the variation among populations (URP = 4%, SCoT = 10%, combined data = 7%). The genetic differentiation coefficient (*G*_*ST*_)/ gene flow (*Nm*) equaled 0.117/3.773, 0.185/2.197, and 0.14/2.86, respectively, for URP, SCoT, and the combined data. Genetic diversity per population varied considerably (Table 4). According to URP data, the highest number of alleles (*Na*) occurred in region I (1.96) and region II (1.94). In region IV and region II, the highest number of effective alleles (*Ne* = 1.60 and 1.58) were detected, as well as the highest value of Nei’s gene diversity (*He* = 0.34) and Shannon’s information (*I* = 0.51). Thus, the highest polymorphic loci (%) (*PPL* = 97.39%) occurred among the population of region I. According to the SCoT results, the populations of region I and region II had the largest *Na* (1.97 and 1.92), *Ne* (1.62 and 1.61), *H* (0.36 and 0.35), and *I* (0.53 and 0.52) values, respectively. Such populations also showed high *PPL* values (98.15 and 94.44%). In addition, region II appeared in the combined data analysis (SCoT + URP) and displayed the highest values for *I* (0.52), *H* (0.35), and *Ne* (1.60). The highest values of these parameters (*PPL* = 97.73%, *Na* = 1.96) occurred in the population of region I.

**Table 3:** Analysis of molecular variance (AMOVA) based on SCoT, URP, and combined data in *Rosa damascena* Mill. populations

Source of variation	URP		SCoT		URP + SCoT	
	Among pops	Within pops	Among pops	Within pops	Among pops	Within pops
*Df*	4	35	4	35	4	35
*SS*	299.88	1982.44	317.37	1492.56	617.24	3475.00
*MS*	74.97	56.64	79.34	42.64	154.31	99.28
*Est.Var*	2.34	56.64	4.68	42.64	7.02	99.28
*Var*	4%	96%	10%	90%	7%	93%
*Phipt*	0.040		0.099		0.066	
*P* = 0.010						
*GST*	0.117		0.185		0.148	
*Nm*	3.773		2.197		2.863	

*Df* degree of freedom, *SS* sum of squares, *MS* mean of squares, *Est. Var* estimated variance components, *Var* total variance, *GST* inter-population differentiation, *Nm* gene flow

**Table 4:** Summary of genetic variation among different Iranian *Rosa damascena* Mill. populations as revealed through URP and SCoT analysis

Marker	Population	*Na*	*Ne*	*I*	*He*	*PPL*
URP	Region I	1.96 ± 0.02	1.55 ± 0.02	0.50 ± 0.01	0.33 ± 0.01	97.39
	Region II	1.94 ± 0.02	1.58 ± 0.02	0.51 ± 0.01	0.34 ± 0.01	96.27
	Region III	1.72 ± 0.04	1.50 ± 0.02	0.45 ± 0.01	0.30 ± 0.01	83.96
	Region IV	1.83 ± 0.03	1.60 ± 0.02	0.51 ± 0.01	0.34 ± 0.01	89.55
	Region V	1.67 ± 0.04	1.47 ± 0.02	0.41 ± 0.02	0.28 ± 0.01	79.85
	Mean	1.823 ± 0.015	1.541 ± 0.009	0.477 ± 0.006	0.318 ± 0.004	89.40 ± 3.40
SCoT	Region I	1.97 ± 0.02	1.62 ± 0.02	0.53 ± 0.01	0.36 ± 0.01	98.15
	Region II	1.92 ± 0.02	1.61 ± 0.02	0.52 ± 0.01	0.35 ± 0.01	94.44
	Region III	1.74 ± 0.04	1.55 ± 0.03	0.45 ± 0.02	0.31 ± 0.01	79.63
	Region IV	1.68 ± 0.05	1.54 ± 0.03	0.45 ± 0.02	0.31 ± 0.01	80.09
	Region V	1.50 ± 0.05	1.43 ± 0.03	0.37 ± 0.02	0.25 ± 0.01	68.06
	Mean	1.76 ± 0.01	1.55 ± 0.01	0.46 ± 0.00	0.31 ± 0.00	84.07 ± 5.47
URP + SCoT	Region I	1.96 ± 0.01	1.58 ± 0.01	0.51 ± 0.01	0.34 ± 0.01	97.73
	Region II	1.93 ± 0.02	1.60 ± 0.01	0.52 ± 0.01	0.35 ± 0.01	95.45
	Region III	1.73 ± 0.03	1.52 ± 0.02	0.45 ± 0.01	0.30 ± 0.01	82.02
	Region IV	1.76 ± 0.03	1.57 ± 0.02	0.48 ± 0.01	0.33 ± 0.01	85.33
	Region V	1.59 ± 0.03	1.45 ± 0.02	0.40 ± 0.01	0.26 ± 0.01	74.59
	Mean	1.80 ± 0.01	1.55 ± 0.01	0.47 ± 0.00	0.32 ± 0.00	87.02 ± 4.29

*Na* observed number of alleles, *Ne* number of effective alleles, *I* Shannon’s information index, *He* Nei’s gene diversity, *PPL* the percentage of polymorphism

### Genetic distances and groping accessions

The Jaccard distance coefficient pairs of accessions were estimated by binary data from URP and SCoT primers. In URP, the genetic distance of a pairwise pattern varied from 0.180 to 0.872 with an average of 0.659 among different pairs of landraces of *Rosa damascena* Mill. The highest distance coefficient (0.872) was observed between accessions Minab (16) (region II) and Barzok1 (9) (region I), whereas the lowest distance (0.180) was identified between two accessions of region V, Khoramabad (39) and Borujerd (40). According to SCoT data, however, the estimated Jaccard’s distance coefficient ranged from 0.107 to 0.785, with an average of 0.602. The largest distance (0.785) was recorded between accessions Barzok2 (11) (region I) and Tabriz (34) (region IV), while the smallest distance (0.107) was observed among two accessions from region III, Semnan1 (26) and Semnan2 (27). The pairwise genetic distance coefficient was measured based on combined data and revealed a wide spectrum of 0.153–0.789, while averaging at 0.631 among all 40 accessions. The largest distance (0.785) was recorded between accessions Barzok2 (11) (region I) and Tabriz (34) (region IV), while the smallest distance (0.107) was observed among two accessions from region III, Semnan1 (26) and Semnan2 (27). The pairwise genetic distance coefficient was identified among Mashhad ardehal (2) and Barzok1 (9) from Esfahan province (region I), whereas the least distance belonged to the Lorestan province (region V) (Khoramabad and Borujerd) (data not shown).

**Fig. 2:**
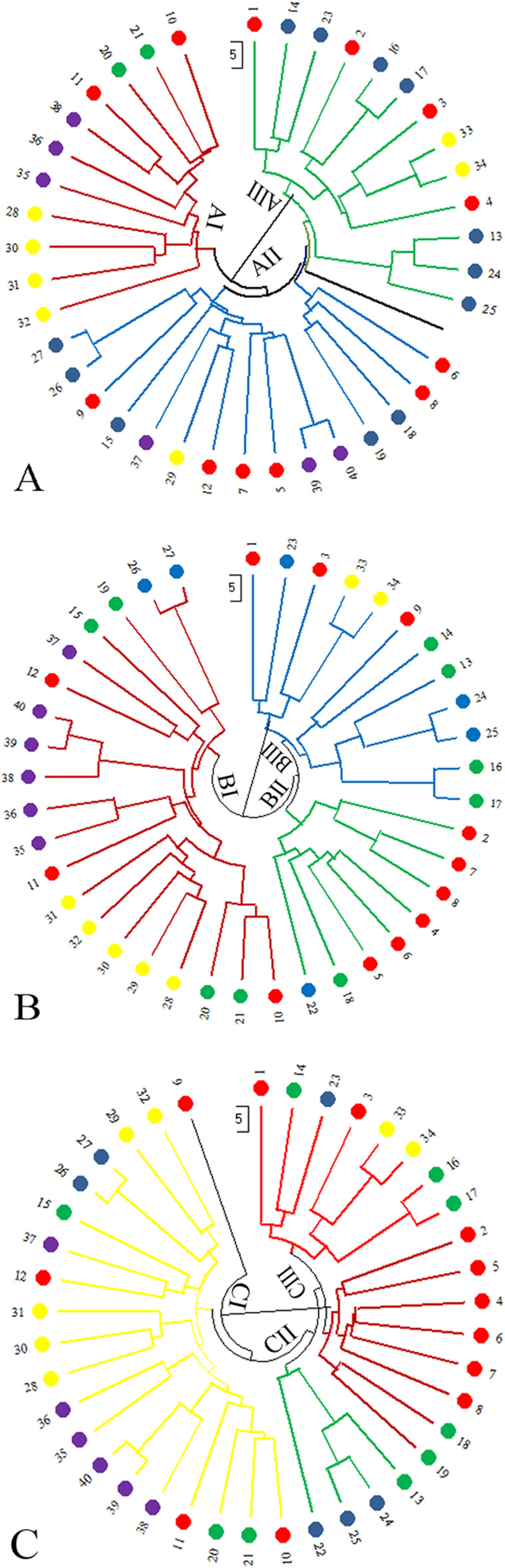
Fan-dendrogram according to the Neighbor-joining method, evaluated with URP (**A**), SCoT (**B**), and SCoT URP (**C**) data. To see the accession codes, refer to Table 1

To examine genetic relationships between genotypes, cluster analysis was used through the Neighbor-joining (NJ) method for the 40 *Rosa damascena* accessions. A dendrogram was created using SCoT, URP, and combined data (SCoT + URP). The dendrogram classified all accessions into three major clusters (Fig. 2A–C). According to the URP data, the first cluster (AI) comprised 11 accessions. The subpopulation AI was divided into clades springing from Gilan (4 accessions), Kermanshah (3 accessions), Esfahan, and Kerman (2 accessions). The second cluster (AII) consisted of 15 accessions from Esfahan (6 accessions), Semnan (2 accessions), Lorestan (2 accessions), Kerman (2 accessions), Gilan, Kermanshah, and Fars (1 accession). Fourteen accessions were classified in the third cluster (AIII) and comprised 4 accessions from Esfahan, 2 accessions from Fars, along with all accessions of Hormozgan, East Azerbaijan, Tehran, and Markazi (Fig. 2A). In SCoT, the cluster BI mainly consisted of 20 accessions from Esfahan, kerman (3 accessions) and all accessions of Gilan, Kermanshah, Lorestan, Semnan, Gilan, Kermanshah, Esfahan, Kerman, Lorestan, and Semnan. The second cluster consisted of Esfahan (6 accessions), Kerman, and Tehran (1 accession) regions (BII). The third cluster (BIII) consisted of 3 accessions from Esfahan, 1 accession of Tehran, along with all accessions of Markazi, Hormozgan, Fars, and East Azerbaijan (Fig. 2B). According to the clustering pattern which was gathered by combined data, the 40 accessions were categorized into three groups (Fig. 2C). The first cluster consisted of 5, 4, 3, 2, 2, 2, and 1 accessions from Gilan, Kermanshah, Esfahan, Kerman, Lorestan, Semnan, and Fars regions, respectively (CI). The second cluster could be separated into sub-originating regions, mainly from Markazi (2 accessions), Tehran, and Fars (1 accession) (CII). The third cluster (CIII) consisted of 9 accessions from Esfahan; 2 accessions from East Azerbaijan, Hormozgan, Kerman; and 1 accession from Tehran and Fars.

Mantel correlation test showed a low and statistically nonsignificant correlation (*r* = 0.49) between distances revealed by SCoT and URP data for all 40 accessions across five collected regions.

### Principal coordinate analyses (PCoA)

**Fig. 3:**
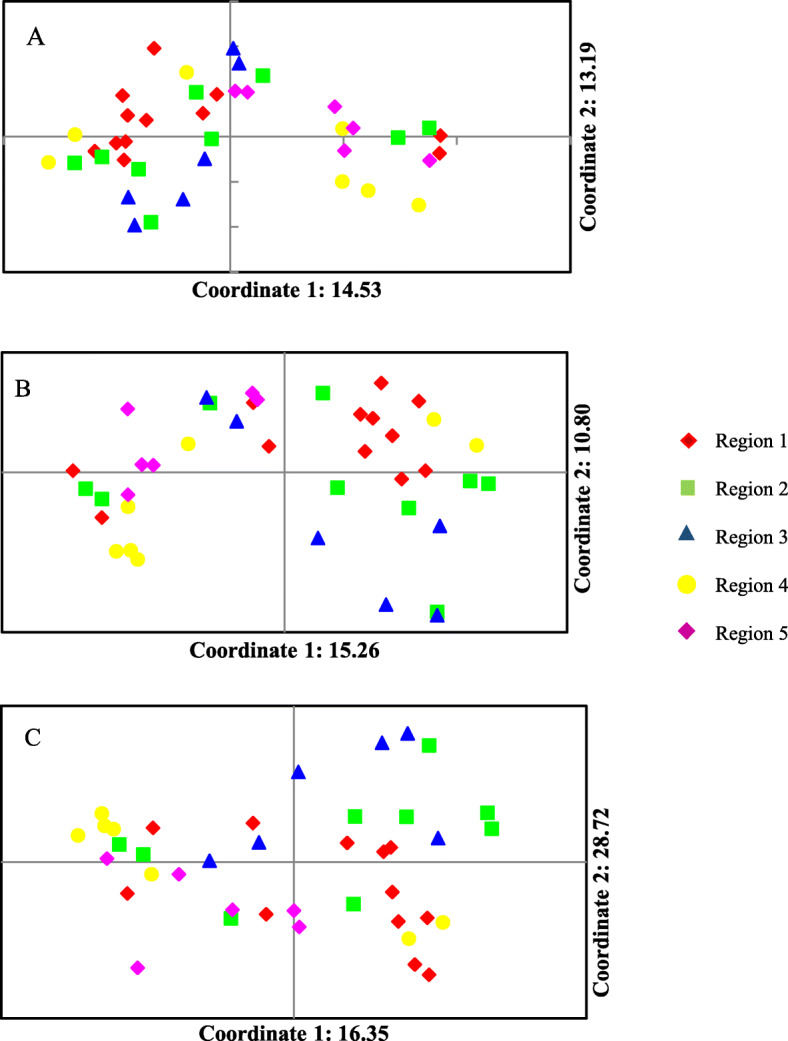
Principal coordinate analyses (PCoA) for 40 Iranian *Rosa damascena* accessions based on URP (**A**), SCoT (**B**), and SCoT URP (**C**) data

The principal coordinate analysis ultimately assisted in analyzing and depicting the population structure. According to URP (A), SCOT (B), and the combined data (C), the first three principal coordinates explained 36.69, 37.34, and 33.85% of molecular variations, respectively. The PCoA biplots showed that all accessions displayed a scattered distribution in the plot, although this did not follow their origins (Fig. 3A–C). Indeed, the results of cluster analysis supported these observations (Fig. 2).

### Population structure analysis

**Fig. 4:**
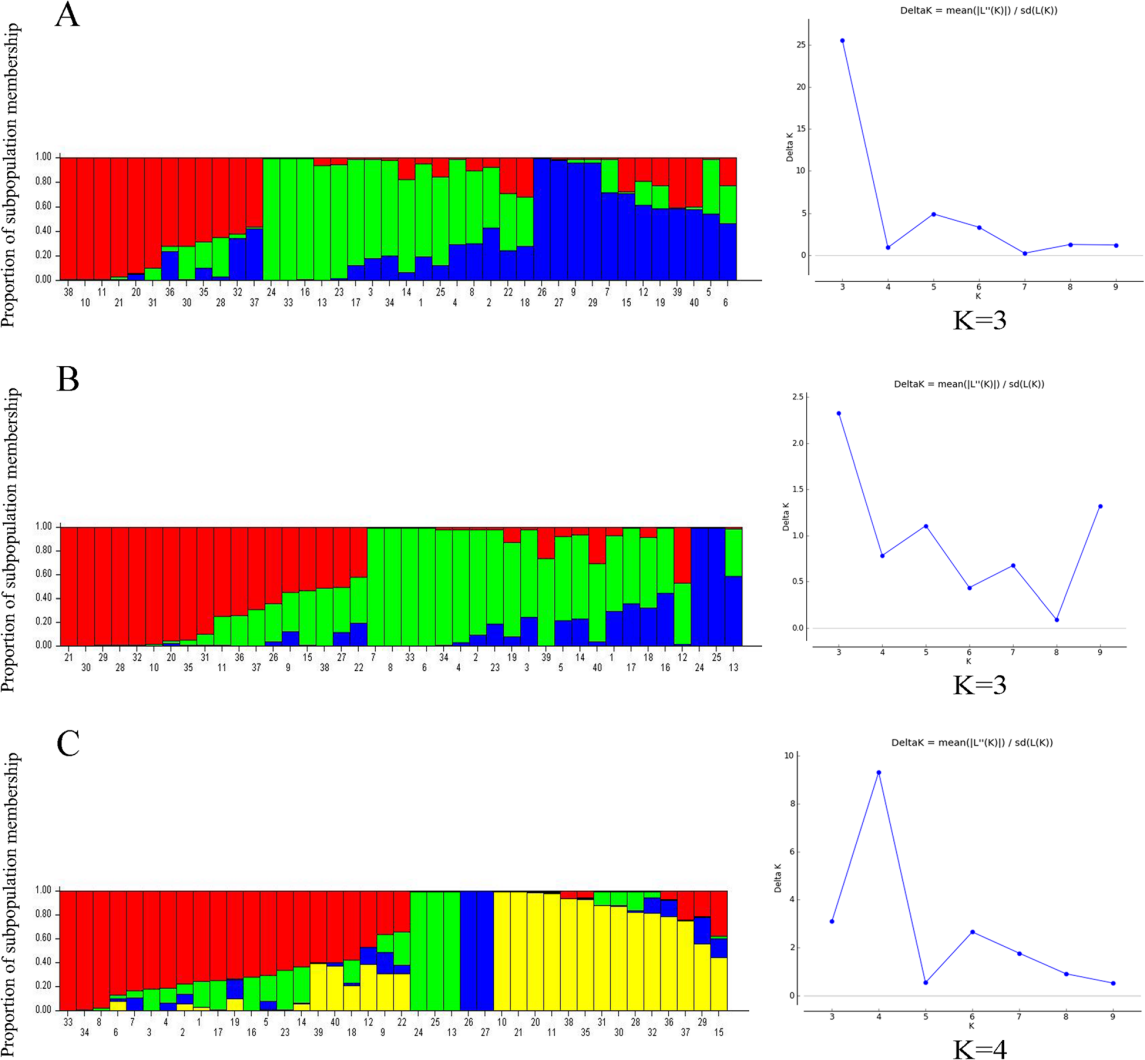
Population structure of 40 *R. damacena* accessions based on URP (A panel), SCoT (B panel), and the combined data (C panel)

Bayesian clustering was used for determining the population structure of the 40 accessions. The membership proportions varied from *K* = 1 to *K* = 10, and with URP primers, probabilities were most precisely derived at *K* = 3. Out of the 40 accessions, subgroup 1 included 12 accessions from Kermanshah (4), Esfahan (2), Gilan (4), and Kerman (2), as well as subgroup 2 which comprised all accessions from Markazi, Tehran, Hormozgan, East Azerbaijan, as well as some accessions from Esfahan (5), Fars (2), and Kerman (1) populations. Twelve accessions of Semnan (2), Lorestan (2), Esfahan (5), Kerman, Gilan, and Fars (1) populations were classified into subgroup 3 (Fig. 4A). As shown in Fig. 4B, delta *K* had the largest ad hoc value at *K* = 3, confirming that the 40 accessions are better divided into three subgroups using SCoT data. Subgroup 1 (17) comprised accessions from Gilan (5), Kermanshah (4), Esfahan (3), Semnan (2), Kerman (2), and Fars (1) populations. Subgroup 2 (15) comprised accessions from the Esfahan (7), Tehran (2), Tabriz (2), Lorestan (2), and Kerman (1) populations; subgroup 3 (8) had accessions from the Esfahan (2), Hormozgan (2), Markazi (2), Fars (1), and Kerman (1) populations. With 26 polymorphic URP and SCoT primers, four different subgroups were achieved (Fig. 4B). Subgroup 1 comprised all accessions from the East Azerbaijan, Tehran, Hormozgan, and Lorestan; 10 accessions from Esfahan; and 2 accessions of Kerman populations, whereas all accessions from the Markazi and one accession from the Fars were grouped into subgroup 2. All accessions from the Semnan were grouped into subgroup 3. Fourteen accessions from Gilan (5), Kermanshah (4), Esfahan (2), Kerman (2), and Fars (1) populations were allocated to subgroup 4.

## Discussion

It is very difficult to evaluate the genetic diversity of *R. damascena* if only morphological features were to be available as markers. Meanwhile, technological tools for the identification of biodiversity include rapid, reliable procedures to describe genetic relationships and variation among roses. DNA markers are the most common tools in current research trends on rose genetic diversity [43–47].

In this research, the genetic variation of the 40 *Rosa damascena* accessions was measured using two marker techniques: URP and SCoT. Our results indicated a significant genetic variation within the populations. We compared the effectiveness of URP and SCoT as new gene-based markers for identifying genetic variation among *Rosa damascena*. By both markers, the proportion of polymorphism turned out to be 100% (Table 2) which was greater than the polymorphic ratios of bands. Given this polymorphic percentage, these markers can serve as a powerful tool in identifying and discriminating between rose genotypes. Henuka et al. [17] used RAPD markers and reported 98.54% polymorphism. Korkmaz and Dogan [21] observed 90.1% and 88.8% polymorphisms among twenty-seven *Rosa spp.* in Turkey, after using ISSR and RAPD markers, respectively. Panwar et al. [18] also reported 94% genetic polymorphism with ISSR markers. Carvalho et al. [48] found 93.7% polymorphism among a selection of rose genotypes based on ISSR markers. Jamali et al. [49] reported 77% polymorphism. These high percentages of polymorphism reflect the heterozygous nature of the polyploid genome structure of rose species. Agarwal et al. [22] studied genetic diversity in 29 Indian rose germplasms using SCoT marker. Based on their results, a high level of polymorphism was observed among the genotypes, which was in line with our results. The SSR markers also not only revealed a high level of diversity in *R. damascena* germplasm in Iran, but also showed a high level of variation in Pakistani genotypes [50].

URP markers showed higher values of *TAB*, *TPB*, *Rp*, *PIC*, and *MI* than SCoT markers in terms of marker informativeness indices. Therefore, the markers showed higher values of these indices and suggested that the Iranian *Rosa damascena* germplasm has a good degree of genetic diversity. The polymorphic information content (*PIC*) of a parameter represents the amount of polymorphism of a marker, as this can vary from zero to half. The larger the value, the greater the number of alleles and the higher the frequency of polymorphisms for that position in the study population. In the present study, the relatively high PIC and MI values for the URP primers provided an estimation of the discriminating ability of the URP marker systems [51]. They showed better resolution and differentiation. In general, in the present experiment, small differences were observed between markers in terms of indices. Statistics showed that both SCoT and URP methods have similar performance in the occurrence of genetic polymorphisms among the evaluated populations. Also, high levels of polymorphism showed that markers of both methods are useful in studying genetic variation. They are equally effective in distinguishing between *Rosa damascena* populations with close kinship ratios.

According to the AMOVA, 96% and 90% of genetic variations were revealed by the URP and SCoT markers, respectively, which were partitioned within populations, suggesting that the observed variation within genotypes was higher than among them (Table 3). Interpopulation differentiation (*GST*) and gene flow (*Nm*) variables backed up these findings. As a result, the *GST* values for URP, SCoT, and combined data were 0.117, 0.185, and 0.148, respectively, revealing that genetic variation among populations is relatively low. The indirect estimate of gene flow (*Nm*) via *GST* was 3.77 (URP), 2.19 (SCoT), and 2.86 (combined data). The total number of migrants per generation exceeds two. Here, genetic differences may be partly due to gene flow, as the populations of this species are significantly affected by genetic drift. Also, local populations are different if *Nm* < 1 [52]. High values of *Nm* occurred in populations, and thus, gene flow prevented drastic genetic differences among gemmates. Population size and the spread of alleles among various regions can add details to this finding [53]. Kiani et al. [45] studied genetic relationships among 41 *R. damascena* accessions from Iran using 31 RAPD. The authors reported that the genetic variation within the collected populations was more than the variation among them. Similar results were achieved in the present study; however, the variation within the populations with both markers was higher than the previous study.

Table 4 shows a list of genotypes of genetic diversity indices. Maximum values of indices in relation to genetic diversity (*Ne*, *Na*, *I*, *PPL*, and *He*) were reported for region I and region II populations using SCoT and combined data. In the URP marker system, region I had the highest polymorphism percentage (*PPL*) and the highest number of alleles (*Na*), whereas precision of genetic diversity was provided by Shannon’s information index and Genetic Diversity Index for populations of regions II and IV. As divergent populations, regions I and II could be selected according to SCoT and combined data, while regions II and IV could be selected according to the URP data. A larger genetic variability here may reflect the population’s frequent allelic variation, while weather conditions can affect ultimate variation among the populations [54]. Furthermore, this finding suggests that these regions could be a strong source of diversity for potential breeding projects which can benefit from new alleles and candidate genes [55]. Also, the highest genetic distance between accessions were based on all marker systems from regions I, II, and IV, as reported in the results of the genetic distance. Therefore, in inbreeding and hybridization systems, these accessions may be used as parents to achieve maximum heterosis if they have desirable traits. According to Pirseyedi et al. [56], an extreme degree of genetic diversity was observed among 12 Iranian Damask rose genotypes [45, 57, 58]. In contrast, Agaoglu et al. [59] and Baydar et al. [44] studied the genetic diversity of *R. damascena* in Turkey, via RAPD and AFLP techniques. Genetic uniformity existed among *R. damascena* cultivars.

In the current study, spatial distribution did not align with genetic relationships, based on the neighbor-joining cluster analysis (Fig. 2). For example, using URP analysis, populations from Iran’s north (Gilan province) and west (Kermanshah province) were grouped together in the same subgroup. Also, with SCoT marker analysis, the populations of Minab (sampled from the south (Hormozgan province)) and Tabriz (sampled from the northwest (East Azerbaijan province)) were classified in the same subgroup. Moreover, the combination of URP and SCoT showed a clustering trend that contradicts the spatial distribution of populations. For example, populations from Esfahan (sampled from the central areas of Iran) and Fars (sampled from the south) were clustered together. Because of the country’s diverse climate and the adaptation of damask rose to adverse environmental conditions, it seems that ecotypes of this plant have been moved and relocated by migrating people across the country, especially on foothills where crops usually do not grow. Pirseyedi et al. [56] noted genetic affinity between the damask rose of Kashan and Kazeroon districts, despite the long distance between them. Baydar et al. [44] used AFLP and microsatellite markers and found that *R. damascena* plants in Turkey can be derived from the same original genotype by vegetative propagation. Rusanov et al. [43] reported that rose plants of Iran and India may have a common origin. Based on the results, the patterns of grouping did not correlate with geographical origin. Similar results were observed in microsatellite analysis of Damask rose accessions from various regions of Iran [55]. Usually, a larger sample is necessary to determine the relationship between molecular data with geographical distance, whether there is isolation of populations due to barriers in gene flow, or whether different climatic conditions lead to differentiation within the species [60].

In the current research, the PCO analysis confirmed the results of cluster analysis. Genetic proximities were visually depicted by PCO among populations. In URP and SCoT, genetic difference and geographical distance were not clear-cut. Besides, phenotypic traits have high correlations in some occasions, while the first two components justify more than 90% of the changes. Meanwhile, molecular markers could not justify the higher values of variance of the primary variables by several of the main components. In investigating the genetic diversity using molecular data, the markers should have a uniform and appropriate distribution in the genome so that they can be sampled from the entire genome. As shown in Fig. 3, the genotypes were well distributed throughout the environment which can be due to the great variety between genotypes and the suitability of markers and primers used. The mini core collection covered a large amount of the genome and had differentiated values among genotypes in the environment.

The neighbor-joining cluster analysis was confirmed by the Bayesian clustering algorithm through STRUCTURE analysis in comparing the 40 accessions [61] (Fig. 4). In the combined data system, however, accessions 27 and 28 (Semnan province) were placed in a separate group (Fig. 4C). Without considering predetermined groups, the Bayesian clustering approach used genetic knowledge to assess the population membership of individuals. Focused on multilocus genotypes, they assign members or parts of their genome to several clusters [62]. Using the online structure harvester software and the Evanno method, the best *K* and the number of subpopulations (∆*K*) were identified. In both marker systems, the best level of population classification were *K* = 3 and in the combined data system *K* = 4. In this clustering, it was found that the different populations of *R. damascena* can group into one cluster, such as cluster ‘BI’ in the SCoT marker system, in which seven populations were grouped from five regions and different altitudes. Moreover, our results showed that clustering by both markers and combined markers made similar classifications of the populations of Kermanshah (region V) and Gilan (region IV), thereby assigning them to the same subgroup, while Hormozgan (region II) and East Azerbaijan (region IV) were classified together in a subgroup. If genotypes or cultivars gather into one category from different areas, it may mean that they have the same genetic heritage [63, 64]. This may have been due to human transmission of plants or genetic movement and displacement by natural variables [65]. The genetic evidence provided here, as well as the available literature, means that plant dispersal by humans has played a large role in the development of *R. damascena* populations throughout Iran. It seems that due to its high tolerance to drought, this crop is one of the most suitable species in arid provinces of the country. Due to a decrease in agricultural water resources and rainfall, its cultivation can replace many agricultural products which have high water requirements. In addition, the ability of this plant to adapt well to different climates and soil conditions in Iran has made farmers inclined to introduce it to other regions in the country. While the genetic origin of these plants is the same in different regions, the obvious difference may be attributed to the climate in which they emerge. Inter and intraspecific variation can be affected by temperature and rainfall [66].

Roses usually cross-pollinate and are self-incompatible which makes them more genetically diverse between and within populations [67–69]. Jurgens et al. [70] investigated the genetic variability of *R. canina* in Brandenburg (Germany). Fifty-five genotypes were classified into twelve subgroups. They attributed the high genetic variation to the outcrossing, seed dispersal system and polyploidy within the *R. canina* populations. The level of genetic variation is affected by breeding system, life cycle, seed dispersal, and geographic distribution which are important factors among populations. Rose species are known to be outcrossing, but there is little evidence on their outcrossing frequencies [71]. Contrary to the results of the current research, a study on *Rosa canina* L. via ISSR markers suggested that geographical distance is effective in causing allelic gaps among genotypes, and ecological conditions could cause genetic variation in *R. canina* [49].

The results of model-based clustering was based on the Bayesian statistical index, assuming that the Ancestry model is Admixture type and the allelic frequency model is of continuous type. While also assuming a range of *K* = 1 to 10 (the number of populations), many populations in the existing germplasm are not completely separated based on the regions from which these genotypes originated or were collected (Fig. 4). The mixing observed in this germplasm confirms the hypothesis that the studied genotypes are of mixed types. That is, plant i may have inherited parts of the genome from offspring in the *K* population. In fact, the formation of different subgroups in population structures depends on the frequency of allelic differences between the genotypes that make up the population. Most of the genotypes were not attributed completely to subgroups, thereby indicating that many genotypes have intermediate genetic traits of various subgroups, as a matter of genetic variation in this research.

## Conclusions

Crop improvement is influenced by information about the degree and distribution of genetic variation, as well as relationships between breeding materials. The results of the present study revealed a high level of polymorphism in the Iranian *R. damascene* populations by the two marker systems. The mean values of PIC for URP and SCoT markers were 0.42 and 0.37, respectively, indicating the efficiency of the two markers in detecting polymorphism among the studied samples. Also, the results confirmed the efficiency of combined data in estimating the genetic diversity among the populations. The used marker systems showed a comprehensive pattern of the genetic diversity among the Iranian *R. damascene* populations, which could provide a future insight into Damask rose breeding programs.

## Supplementary Information

**Additional file 1: Supplementary Table1.** Genotype classification. Classification of genotypes into different groups according to the figure 2 based on SCoT +URP (C) data

**Additional file 2: Supplementary Table2.** Pairwise genetic distance coefficients. Jaccard distance coefficients based on SCoT and URP data

**Additional file 3: Supplementary figure1.** Mantel correlation. The correlation between SCoT and URP data for all 40 accessions across five regions

## Data Availability

All data generated or analyzed during this study are included in this published article.

## Abbreviations

URP: Universal rice primer
SCoT: Start codon targeted
MI: Marker index
PIC: Polymorphism information content
Rp: Resolving power
Na: Number of observed alleles
Ne: Number of effective alleles
He: Nei’s gene diversity
NPB: Number of polymorphic bands
PCoA: Principal coordinate analysis
I: Shannon’s information index
TAB: Total amplified bands
PPB: Percentage of polymorphism
Ta: Temperature annealing
AMOVA: Analysis of molecular variance
Df: Degree of freedom
SS: Sum of squares
MS: Mean of squares
Est. Var: Estimated variance components
Var: Total variance

## References

[1] Gudin S (2000). Rose genetics and breeding. Plant Breed Rev.

[2] Chevallier A (1996). The Encyclopaedia of Medicinal Plants.

[3] Kovats E (1987). Composition of essential oil Part 7 Bulgarian oil of rose (*Rosa damascene* Mill.) J. Chromatogr.

[4] Mahmoud N, Piacente S, Pizza C, Bueke A, Khan AI, Hay AJ (1996). The anti-HIV activity and mechanisms of action of pure compounds isolated from *Rosa damascene*. Biochem Biophys Res Commun.

[5] Andogan BC, Baydar H, Kaya S, Demirci M, Ozbasar D, Mumcu E (2002). Antimicrobial activity and chemical composition of some essential oils. Arch Pharm Res.

[6] Ozkan G, Sagdic O, Baydar NG, Baydar H (2004). Antioxidant and anti-bacterial activities of *R. damascena* flower extracts. Food Sci Technol Int.

[7] Lewis WH (1957). Revision of the genus Rosa in Eastern North America: a review. American Rose Annual.

[8] Kiani M, Zamani Z, Khalighi A, Fatahi R, Byrne DH (2010). Microsatellite analysis of Iranian Damask rose (*Rosa damascena* Mill.) germplasm. Plant Breed.

[9] Kuhns LJ, Fretz TA (1978). Distinguishing rose cultivars by polyacrylamide gel electrophoresis. Isozyme variation among cultivars. J Am Soc Hortic Sci.

[10] Lee JS, Kim YR (1982). Genetic studies on natural populations of *Rosa multiflora* Thunb. by isozyme and multivariate analyses (Korean). Hanguk Wonye Hakhoe Chi.

[11] Walker CA, Werner DJ (1997). Isozyme and randomly amplified polymorphic DNA (RAPD) analyses of Cherokee rose and its putative hybrids ‘Silver Moon’ and ‘Anemone’. J Am Soc Hortic Sci.

[12] Kim Y (1994) A study of selected species of Rosa using isozyme polymorphisms. M Sc thesis, Texas A and M University, College Station, Texas. DOI:10.21273/HORTSCI.34.2.341

[13] Williams JGK, Kubelik AR, Livak KJ, Rafalski JA, Tingey SV (1990). DNA polymorphisms amplified by arbitrary primers are useful as genetic markers. Nucleic Acids Res.

[14] Mirali N, Aziz R, Nabulsi I (2012). Genetic characterization of *Rosa damascene* species growing in different regions of Syria and its relationship to the quality of the essential oils. Int J Med Arom Plants.

[15] Azeem S, Khan AI, Awan FS, Riaz A, Bahadur S (2012). Genetic diversity of rose germplasm in Pakistan characterized by random amplified polymorphic DNA (RAPD) markers. Afr J Biotechnol.

[16] Vukosavljev M, Zhang J, Esselink GDWPC, van’t Westende CP, Visser RGF, Arens P, Smulders MJM (2013). Genetic diversity and differentiation in roses: a garden rose perspective. Sci Hortic (Amsterdam).

[17] Henuka R, Raju D, Janakiram N (2015). Characterization and analysis of genetic diversity among different species of rose (*Rosa* species) using morphological and molecular markers. Indian J Agric Sci.

[18] Panwar S, Singh KP, Sonah H, Deshmukh R, Prasad K, Sharma T (2015). Molecular fingerprinting and assessment of genetic diversity in rose (*Rosa* × hybrida). Indian J Biotechnol.

[19] Rai H, Raju D, Kumar A, Janakiram T, Namita S, Krishnan G, Rana J (2015). Characterization and analysis of genetic diversity among different species of the rose using morphological and molecular markers. Indian J Agr Sci.

[20] Ogras T, Bastanlar EK, Metin ÖK, Kandemir I, Özcelik H (2017). Assessment of genetic diversity of rose genotypes using ISSR markers. Turk J Botany.

[21] Korkmaz M, Dogan NY (2018). Analysis of genetic relationships between wild roses (*Rosa* L. Spp.) growing in Turkey. Erwerbs-Obstbau.

[22] Agarwal A, Gupta V, Haq SU, Jatav PK, Kothari S, Kachhwaha S (2019). Assessment of genetic diversity in 29 rose germplasms using SCoT marker. J King Saud Univ Sci.

[23] Collard BC, Mackill DJ (2009). Start codon targeted (SCoT) polymorphism: a simple, novel DNA marker technique for generating gene-targeted markers in plants. Plant Mol Biol Report.

[24] Amom T, Nongdam P (2017). The use of molecular marker methods in plants: a review. Int J Curr Res Rev.

[25] Gorji AM, Poczai P, Polgar Z, Taller J (2011). Efficiency of arbitrarily amplified dominant markers (SCoT, ISSR and RAPD) for diagnostic fingerprinting in tetraploid potato. Am J Potato Res.

[26] Zhang J, Xie W, Wang Y, Zhao X (2015). Potential of start codon targeted (SCoT) markers to estimate genetic diversity and relationships among Chinese *Elymus sibiricus* accessions. Molecules.

[27] Etminan A, Pour-Aboughadareh A, Noori A, Ahmadi-Rad A, Shooshtari L, Mahdavian Z, Yousefiazar-Khanian M (2018). Genetic relationships and diversity among wild Salvia accessions revealed by ISSR and SCoT markers. Biotechnol Biotechnol Equip.

[28] Jalilian H, Zarei A, Erfani-Moghadam J (2018). Phylogeny relationship among commercial and wild pear species based on morphological characteristics and SCoT molecular markers. Sci Hortic.

[29] Saidi A, Daneshvar Z, Hajibarat Z (2018). Comparison of genetic variation of anthurium (Anthurium andraeanum) cultivars using SCoT, CDDP and RAPD markers. Plant Tissue Cult Biotechnol.

[30] Amom T, Tikendra L, Apana N, Goutam M, Sonia P, Koijam AS, Nongdam P (2020). Efficiency of RAPD, ISSR, iPBS, SCoT and phytochemical markers in the genetic relationship study of five native and economical important bamboos of North-East India. Phytochemistry.

[31] Kang H, Kwon S, Go S (2003). PCR-based specific and sensitive detection of *Pectobacterium carotovorum* ssp. carotovorum by primers generated from a URP-PCR fingerprinting-derived polymorphic band. Plant Pathol.

[32] Powell W, Morgante M, Andre C, Hanafey M, Vogel J, Tingey S, Rafalski A (1996) The comparison of RFLP, RAPD, AFLP and SSR (microsatellite) markers for germplasm analysis. Mol Breed 2(3), 225-238. DOI. 10.1007/BF00564200

[33] Doyle JJ, Doyle JL (1990). Isolation of plant DNA from fresh tissue. Focus.

[34] Singh AK, Rana M, Singh S, Kumar S, Kumar R, Singh R (2014). CAAT box-derived polymorphism (CBDP): a novel promoter-targeted molecular marker for plants. J Plant Biochem Biotechnol.

[35] Anderson JA, Churchill G, Autrique J, Tanksley S, Sorrells M (1993). Optimizing parental selection for genetic linkage maps. Genome.

[36] Peakall R, Smouse PE (2006). GENALEX 6: genetic analysis in Excel. Population genetic software for teaching and research. Mol Ecol Notes.

[37] Kimura M, Crow JF (1964). The number of alleles that can be maintained in a finite population. Genetics.

[38] Nei M (1978). Estimation of average heterozygosity and genetic distance from a small number of individuals. Genetics.

[39] Lewontin RC (1972) The apportionment of human diversity. Evolutionary Biology:381–398. 10.1007/978-1-4684-9063-3_14

[40] Jaccard P (1908). Nouvelles recherches sur la distribution florale. Bull Soc Vaud Sci Nat.

[41] Tamura K, Peterson D, Peterson N, Stecher G, Nei M, Kumar S (2011). MEGA5: molecular evolutionary genetics analysis using maximum likelihood, evolutionary distance, and maximum parsimony methods. Mol Biol Evol.

[42] Pritchard JK, Stephens M, Donnelly P (2000). Inference of population structure using multilocus genotype data. Genetics.

[43] Rusanov K, Kovacheva N, Vosman B, Zhang L, Rajapakse S, Atanassov A, Atanassov I (2005). Microsatellite analysis of *Rosa damascena* Mill. accessions reveals genetic similarity between genotypes used for rose oil production and old Damask rose varieties. Theor Appl Genet.

[44] Baydar NG, Baydar H, Debener T (2004). Analysis of genetic relationships among *Rosa damascena* plants grown in Turkey by using AFLP and microsatellite markers. J Biotechnol.

[45] Kiani M, Zamani Z, Khalighi A, Fatahi R, Byrne DH (2008). Wide genetic diversity of *Rosa damascena* Mill. germplasm in Iran as revealed by RAPD analysis. Sci Hortic.

[46] Koopman WJ, Wissemann V, De Cock K, Van Huylenbroeck J, De Riek J, Sabatino GJ, Maes B (2008). AFLP markers as a tool to reconstruct complex relationships: a case study in *Rosa* (Rosaceae). Am J Bot.

[47] Yan Z, Denneboom C, Hattendorf A, Dolstra O, Debener T, Stam P, Visser P (2005). Construction of an integrated map of rose with AFLP, SSR, PK, RGA, RFLP, SCAR and morphological markers. Theor Appl Genet.

[48] Carvalho A, Lima-Brito J, Macas B, Guedes-Pinto H (2009). Genetic diversity and variation among botanical Portuguese wheat cultivars revealed by ISSR assays. Biochem Genet.

[49] Jamali M, Ghanbari A, Estaji A, Torabi Giglou M, Saidi M (2019). Genetic diversity of dog rose (Rosa canina L.) using ISSR markers. Iran J Genet Plant Breed.

[50] Farooq A, Kiani M, Khan MA, Riaz A, Khan AA, Anderson N, Byrne DH (2013). Microsatellite analysis of *Rosa damascena* from Pakistan and Iran. Hortic Environ Biotechnol.

[51] Hajmansoor S, Bihamta MR, Alisoltani A (2013). Genetic diversity among and within Iranian and non-Iranian barely (*Hordeum vulgare* L.) genotypes using SSR and storage proteins markers. Biochem Syst Ecol.

[52] Wright S (1951). The genetical structure of populations. Ann Eugen.

[53] Dumolin-Lapegue S, Demesure B, Fineschi S, Le Corre V, Petit RJ (1997). Phylogeographic structure of white oaks throughout the European continent. Genetics.

[54] Ni J-L, Zhu AG, Wang XF, Xu Y, Sun ZM, Chen JH, Luan MB (2018). Genetic diversity and population structure of ramie (*Boehmeria nivea* L). Ind Crops Prod.

[55] Gholamian F, Etminan A, Changizi M, Khaghani S, Gomarian M (2019). Assessment of genetic diversity in Triticum urartu Thumanjan ex Gandilyan accessions using start codon targeted polymorphism (SCoT) and CAAT-box derived polymorphism (CBDP) markers. Biotechnol Biotechnol Equip.

[56] Pirseyedi SM, Mardi M, Davazdahemami S, Kermani MJ, Mohammadi SA (2005). Analysis of the genetic diversity of 12 Iranian Damask rose (*Rosa damascena* Mill.) genotypes using amplified fragment length polymorphism markers. Iran J Biotech.

[57] Babaei A, Tabaei-Aghdaei SR, Khosh-Khui M, Omidbaigi R, Naghavi MR, Esselink GD, Smulders MJ (2007). Microsatellite analysis of Damask rose (*Rosa damascena* Mill.) accessions from various regions in Iran reveals multiple genotypes. BMC Plant Biology.

[58] Mirzaei L, Rahmani F, and Beigmohamadi M (2015) Assessment of genetic variation among *Rosa* species using ISSR genetic marker. J Biodivers Environ Sci 7(3), 254_260.https://www.researchgate.net/publication/282504457_ASSESMENT_OF_GENETIC_VARIATION_AMONG_ROSA_SPECIES_USING_ISSR_GENETIC_MARKERS

[59] Agaoglu Y, Ergül A, Baydar N (2000). Molecular analysis of genetic diversity oil rose (*Rosa damascena* Mill.) grown Isparta (Turkey) region. Biotechnol Biotechnol Equip.

[60] Goudarzi F, Hemami MR, Rancilhac L, Malekian M, Fakheran S, Elmer KR, Steinfartz S (2019). Geographic separation and genetic differentiation of populations are not coupled with niche differentiation in threatened Kaiser’s spotted newt (Neurergus kaiseri). Scientific Reports.

[61] Evanno G, Regnaut S, Goudet J (2005). Detecting the number of clusters of individuals using the software STRUCTURE: a simulation study. Mol Ecol.

[62] Chen C, Durand E, Forbes F, François O (2007). Bayesian clustering algorithms ascertaining spatial population structure: a new computer program and a comparison study. Mol Ecol Notes.

[63] Besnard G, Khadari B, Villemur P, Bervillé A (2000). Cytoplasmic male sterility in the olive (*Olea europaea* L). Theor Appl Genet.

[64] Sarri V, Baldoni L, Porceddu A, Cultrera N, Contento A, Frediani M, Cionini P (2006). Microsatellite markers are powerful tools for discriminating among olive cultivars and assigning them to geographically defined populations. Genome.

[65] Percifield RJ, Hawkins JS, McCoy JA, Widrlechner MP, Wendel JF (2007). Genetic diversity in Hypericum and AFLP markers for species-specific identification of *H. perforatum* L. Planta Med.

[66] Nybom H, Carlson-Nilsson U, Werlemark G, Uggla M (1997). Different levels of morphometric variation in three heterogamous dog rose species (*Rosa* sect. *Caninae,* Rosaceae). Plant Syst Evol.

[67] Cole P, and Melton B (1986) Self-and cross-compatibility relationships among genotypes and between ploidy of the rose. J Am Soc Hortic Sci 111(1), 122-125.

[68] Ueda Y, Akimoto S (2001). Cross-and self-compatibility in various species of the genus Rosa. J Hortic Sci Biotechnol.

[69] Charlesworth D (2003). Effects of inbreeding on the genetic diversity of populations. Philos Trans R Soc Lond B Biol Sci.

[70] Jürgens R, Ball A, Verster A (2009). Interventions to reduce HIV transmission related to injecting drug use in prison. Lancet Infect Dis.

[71] Jürgens A, Seitz B, Kowarik I (2007). Genetic differentiation of *Rosa canina* (L.) at regional and continental scales. Plant Syst Evol.

