# Supplementary material for: Genetic diversity analysis in a mini core collection of Damask rose (Rosa damascena Mill.) germplasm from Iran using URP and SCoT markers
Source: J Genet Eng Biotechnol. 2021 Sep 30;19:144. doi: 10.1186/s43141-021-00247-7 (PMC8484433; doi:10.1186/s43141-021-00247-7)
Supplement: Supplementary file 1 — Additional file 1: Supplementary Table1. Genotype classification. Classification of genotypes into different groups according to the figure 2 based on SCoT +URP (C) data [file 43141_2021_247_MOESM1_ESM.docx]

| **Table S1:** classified genotypes into different groups according to the figure 2 based on SCoT +URP (C) data. | | | | |
| --- | --- | --- | --- | --- |
| NO | Accession | Region | Province | cluster |
| 10 | Kamu2 | I | Esfahan | 1 |
| 21 | Sirach | III | Kerman | 1 |
| 20 | kerman | III | Kerman | 1 |
| 11 | Barzok2 | I | Esfahan | 1 |
| 38 | Koohdasht2 | V | Kermanshah | 1 |
| 39 | Khoramabad | V | Lorestan | 1 |
| 40 | Borujerd | V | Lorestan | 1 |
| 35 | Gareban1 | V | Kermanshah | 1 |
| 36 | Gareban2 | V | Kermanshah | 1 |
| 28 | lahijan | IV | Gilan | 1 |
| 30 | Chaboksar | IV | Gilan | 1 |
| 31 | Astaneh | IV | Gilan | 1 |
| 12 | Barzok3 | I | Esfahan | 1 |
| 37 | Koohdasht1 | V | Kermanshah | 1 |
| 15 | Shiraz | II | Fars | 1 |
| 26 | Semnan1 | III | Semnan | 1 |
| 27 | Semnan2 | III | Semnan | 1 |
| 29 | Rasht | IV | Gilan | 1 |
| 32 | Somesara | IV | Gilan | 1 |
| 13 | Darab | II | Fars | 2 |
| 24 | Ghalhar | III | Markazi | 2 |
| 25 | Delijan | III | Markazi | 2 |
| 22 | Firuzkuh | III | Tehran | 2 |
| 1 | Ghamsar1 | I | Esfahan | 3 |
| 14 | Meymand | II | Fars | 3 |
| 23 | Lavasanat | III | Tehran | 3 |
| 3 | Kamu1 | I | Esfahan | 3 |
| 33 | Tabriz1 | IV | East Azerbijan | 3 |
| 34 | Tabriz2 | IV | East Azerbijan | 3 |
| 16 | Minab | II | Hormozgan | 3 |
| 17 | Hormozgan | II | Hormozgan | 3 |
| 2 | Ardehal | I | Esfahan | 3 |
| 5 | Ozvar | I | Esfahan | 3 |
| 4 | Azeran | I | Esfahan | 3 |
| 6 | Ghamsar2 | I | Esfahan | 3 |
| 7 | Nabar | I | Esfahan | 3 |
| 8 | Ghohrud | I | Esfahan | 3 |
| 18 | Bardsir | II | Kerman | 3 |
| 9 | Barzok1 | I | Esfahan | 3 |
| 19 | Lalezar | III | Kerman | 3 |
