# Supplementary material for: Genetic diversity analysis in a mini core collection of Damask rose (Rosa damascena Mill.) germplasm from Iran using URP and SCoT markers
Source: J Genet Eng Biotechnol. 2021 Sep 30;19:144. doi: 10.1186/s43141-021-00247-7 (PMC8484433; doi:10.1186/s43141-021-00247-7)
Supplement: Supplementary file 2 — Additional file 2: Supplementary Table2. Pairwise genetic distance coefficients. Jaccard distance coefficients based on SCoT and URP data [file 43141_2021_247_MOESM2_ESM.docx]

| **Table S2-1:** The pairwise genetic distance coefficients estimated based on Jaccard distance coefficients based on **SCoT** data | | | | | | | | | | | | | | | | | | |
| --- | --- | --- | --- | --- | --- | --- | --- | --- | --- | --- | --- | --- | --- | --- | --- | --- | --- | --- |
| Genotypes | | distance |  | Genotypes | | distance |  | Genotypes | | distance |  | Genotypes | | distance |  | Genotypes | | distance |
| Id(i) | Id(j) | d(i,j) |  | Id(i) | Id(j) | d(i,j) |  | Id(i) | Id(j) | d(i,j) |  | Id(i) | Id(j) | d(i,j) |  | Id(i) | Id(j) | d(i,j) |
| 34 | 11 | 0.786 |  | 37 | 33 | 0.725 |  | 33 | 30 | 0.696 |  | 34 | 21 | 0.681 |  | 14 | 2 | 0.662 |
| 15 | 8 | 0.784 |  | 34 | 26 | 0.725 |  | 31 | 17 | 0.696 |  | 12 | 9 | 0.680 |  | 37 | 31 | 0.661 |
| 21 | 8 | 0.779 |  | 31 | 2 | 0.725 |  | 16 | 11 | 0.696 |  | 33 | 32 | 0.679 |  | 24 | 7 | 0.660 |
| 37 | 8 | 0.778 |  | 37 | 16 | 0.723 |  | 37 | 3 | 0.695 |  | 34 | 31 | 0.679 |  | 27 | 14 | 0.660 |
| 31 | 8 | 0.776 |  | 24 | 8 | 0.723 |  | 36 | 34 | 0.695 |  | 40 | 11 | 0.679 |  | 23 | 2 | 0.660 |
| 33 | 11 | 0.775 |  | 3 | 2 | 0.723 |  | 37 | 9 | 0.695 |  | 32 | 8 | 0.678 |  | 32 | 2 | 0.660 |
| 15 | 6 | 0.764 |  | 15 | 7 | 0.723 |  | 19 | 15 | 0.694 |  | 28 | 15 | 0.678 |  | 29 | 2 | 0.660 |
| 20 | 8 | 0.762 |  | 21 | 2 | 0.723 |  | 33 | 27 | 0.694 |  | 35 | 7 | 0.677 |  | 19 | 14 | 0.660 |
| 37 | 2 | 0.761 |  | 29 | 8 | 0.722 |  | 25 | 15 | 0.692 |  | 34 | 25 | 0.677 |  | 36 | 33 | 0.660 |
| 28 | 8 | 0.761 |  | 27 | 8 | 0.721 |  | 27 | 2 | 0.692 |  | 31 | 16 | 0.677 |  | 32 | 15 | 0.660 |
| 9 | 2 | 0.758 |  | 34 | 27 | 0.721 |  | 34 | 24 | 0.691 |  | 25 | 11 | 0.677 |  | 23 | 8 | 0.660 |
| 11 | 2 | 0.755 |  | 31 | 12 | 0.721 |  | 31 | 7 | 0.691 |  | 15 | 5 | 0.675 |  | 26 | 6 | 0.658 |
| 34 | 15 | 0.752 |  | 9 | 7 | 0.720 |  | 33 | 8 | 0.691 |  | 34 | 10 | 0.675 |  | 17 | 7 | 0.658 |
| 37 | 24 | 0.749 |  | 14 | 11 | 0.717 |  | 15 | 11 | 0.690 |  | 25 | 2 | 0.675 |  | 21 | 19 | 0.658 |
| 37 | 34 | 0.746 |  | 40 | 15 | 0.715 |  | 13 | 8 | 0.690 |  | 39 | 8 | 0.674 |  | 14 | 7 | 0.658 |
| 12 | 8 | 0.745 |  | 36 | 8 | 0.712 |  | 15 | 13 | 0.690 |  | 19 | 2 | 0.674 |  | 37 | 1 | 0.657 |
| 34 | 8 | 0.744 |  | 37 | 26 | 0.710 |  | 17 | 15 | 0.689 |  | 8 | 3 | 0.674 |  | 28 | 17 | 0.657 |
| 37 | 25 | 0.744 |  | 28 | 2 | 0.710 |  | 34 | 22 | 0.689 |  | 37 | 4 | 0.673 |  | 32 | 17 | 0.657 |
| 23 | 11 | 0.743 |  | 35 | 34 | 0.710 |  | 40 | 34 | 0.688 |  | 17 | 11 | 0.673 |  | 39 | 34 | 0.657 |
| 11 | 8 | 0.742 |  | 21 | 7 | 0.709 |  | 34 | 12 | 0.688 |  | 33 | 18 | 0.673 |  | 24 | 2 | 0.656 |
| 37 | 27 | 0.741 |  | 11 | 1 | 0.708 |  | 21 | 15 | 0.688 |  | 32 | 7 | 0.673 |  | 35 | 2 | 0.656 |
| 11 | 7 | 0.741 |  | 37 | 7 | 0.708 |  | 37 | 5 | 0.688 |  | 28 | 7 | 0.673 |  | 31 | 6 | 0.655 |
| 31 | 14 | 0.739 |  | 17 | 8 | 0.707 |  | 34 | 32 | 0.688 |  | 20 | 15 | 0.671 |  | 19 | 12 | 0.655 |
| 34 | 2 | 0.739 |  | 26 | 8 | 0.707 |  | 34 | 29 | 0.688 |  | 20 | 2 | 0.671 |  | 11 | 9 | 0.655 |
| 37 | 14 | 0.738 |  | 34 | 18 | 0.707 |  | 12 | 11 | 0.688 |  | 17 | 2 | 0.671 |  | 18 | 12 | 0.654 |
| 9 | 8 | 0.738 |  | 34 | 7 | 0.705 |  | 26 | 2 | 0.687 |  | 33 | 29 | 0.671 |  | 33 | 28 | 0.654 |
| 11 | 6 | 0.737 |  | 40 | 2 | 0.704 |  | 16 | 8 | 0.687 |  | 25 | 7 | 0.671 |  | 33 | 7 | 0.654 |
| 37 | 17 | 0.735 |  | 11 | 4 | 0.704 |  | 16 | 15 | 0.686 |  | 22 | 15 | 0.671 |  | 40 | 14 | 0.654 |
| 34 | 14 | 0.735 |  | 23 | 15 | 0.703 |  | 39 | 11 | 0.686 |  | 35 | 6 | 0.671 |  | 34 | 28 | 0.654 |
| 37 | 18 | 0.734 |  | 20 | 7 | 0.702 |  | 19 | 11 | 0.686 |  | 33 | 31 | 0.669 |  | 19 | 8 | 0.654 |
| 11 | 3 | 0.733 |  | 33 | 15 | 0.701 |  | 37 | 11 | 0.685 |  | 31 | 11 | 0.669 |  | 39 | 27 | 0.653 |
| 10 | 8 | 0.731 |  | 33 | 9 | 0.701 |  | 27 | 11 | 0.685 |  | 40 | 33 | 0.669 |  | 35 | 17 | 0.653 |
| 18 | 15 | 0.731 |  | 35 | 33 | 0.701 |  | 8 | 5 | 0.685 |  | 27 | 7 | 0.669 |  | 38 | 2 | 0.653 |
| 30 | 8 | 0.730 |  | 33 | 20 | 0.700 |  | 39 | 15 | 0.684 |  | 27 | 9 | 0.669 |  | 33 | 21 | 0.652 |
| 15 | 2 | 0.729 |  | 15 | 14 | 0.699 |  | 27 | 15 | 0.683 |  | 9 | 6 | 0.669 |  | 27 | 17 | 0.652 |
| 33 | 2 | 0.728 |  | 8 | 1 | 0.699 |  | 37 | 15 | 0.683 |  | 12 | 2 | 0.667 |  | 23 | 12 | 0.652 |
| 34 | 9 | 0.728 |  | 37 | 22 | 0.699 |  | 34 | 13 | 0.683 |  | 22 | 11 | 0.665 |  | 13 | 7 | 0.652 |
| 22 | 8 | 0.727 |  | 37 | 23 | 0.699 |  | 31 | 19 | 0.683 |  | 26 | 14 | 0.665 |  | 33 | 25 | 0.652 |
| 30 | 2 | 0.727 |  | 15 | 1 | 0.699 |  | 24 | 11 | 0.683 |  | 26 | 9 | 0.665 |  | 31 | 23 | 0.652 |
| 35 | 8 | 0.727 |  | 37 | 19 | 0.699 |  | 38 | 34 | 0.682 |  | 40 | 27 | 0.664 |  | 31 | 4 | 0.652 |
| 34 | 20 | 0.727 |  | 33 | 26 | 0.698 |  | 30 | 7 | 0.682 |  | 31 | 27 | 0.664 |  | 40 | 37 | 0.652 |
| 25 | 8 | 0.726 |  | 37 | 6 | 0.697 |  | 24 | 15 | 0.682 |  | 39 | 2 | 0.664 |  | 30 | 6 | 0.651 |
| 40 | 8 | 0.726 |  | 33 | 12 | 0.697 |  | 18 | 11 | 0.682 |  | 35 | 19 | 0.663 |  | 20 | 17 | 0.651 |
| 37 | 13 | 0.726 |  | 34 | 30 | 0.697 |  | 14 | 8 | 0.681 |  | 34 | 4 | 0.662 |  | 33 | 24 | 0.650 |

| **Table S2-1:** The pairwise genetic distance coefficients estimated based on Jaccard distance coefficients based on **SCoT** data | | | | | | | | | | | | | | | | | | |
| --- | --- | --- | --- | --- | --- | --- | --- | --- | --- | --- | --- | --- | --- | --- | --- | --- | --- | --- |
| Genotypes | | distance |  | Genotypes | | distance |  | Genotypes | | distance |  | Genotypes | | distance |  | Genotypes | | distance |
| Id(i) | Id(j) | d(i,j) |  | Id(i) | Id(j) | d(i,j) |  | Id(i) | Id(j) | d(i,j) |  | Id(i) | Id(j) | d(i,j) |  | Id(i) | Id(j) | d(i,j) |
| 13 | 11 | 0.650 |  | 35 | 15 | 0.636 |  | 20 | 14 | 0.624 |  | 2 | 1 | 0.615 |  | 31 | 5 | 0.605 |
| 26 | 15 | 0.650 |  | 26 | 7 | 0.636 |  | 40 | 26 | 0.624 |  | 39 | 25 | 0.615 |  | 19 | 5 | 0.605 |
| 26 | 17 | 0.648 |  | 40 | 9 | 0.635 |  | 20 | 19 | 0.624 |  | 25 | 19 | 0.615 |  | 35 | 4 | 0.605 |
| 21 | 1 | 0.648 |  | 20 | 6 | 0.635 |  | 18 | 8 | 0.624 |  | 26 | 23 | 0.614 |  | 34 | 19 | 0.605 |
| 10 | 2 | 0.648 |  | 37 | 21 | 0.634 |  | 40 | 22 | 0.624 |  | 39 | 9 | 0.614 |  | 39 | 31 | 0.604 |
| 34 | 6 | 0.647 |  | 26 | 3 | 0.634 |  | 24 | 6 | 0.624 |  | 15 | 4 | 0.614 |  | 21 | 6 | 0.604 |
| 23 | 7 | 0.647 |  | 25 | 12 | 0.634 |  | 19 | 9 | 0.623 |  | 27 | 21 | 0.613 |  | 35 | 12 | 0.604 |
| 31 | 3 | 0.647 |  | 16 | 2 | 0.634 |  | 31 | 15 | 0.623 |  | 22 | 19 | 0.613 |  | 26 | 20 | 0.604 |
| 29 | 7 | 0.647 |  | 29 | 17 | 0.634 |  | 36 | 6 | 0.623 |  | 40 | 3 | 0.613 |  | 36 | 14 | 0.604 |
| 15 | 12 | 0.646 |  | 28 | 14 | 0.633 |  | 31 | 24 | 0.623 |  | 13 | 2 | 0.613 |  | 8 | 4 | 0.603 |
| 36 | 9 | 0.646 |  | 25 | 6 | 0.633 |  | 31 | 13 | 0.623 |  | 22 | 2 | 0.613 |  | 35 | 14 | 0.602 |
| 20 | 11 | 0.646 |  | 38 | 15 | 0.633 |  | 40 | 7 | 0.622 |  | 36 | 31 | 0.612 |  | 24 | 21 | 0.602 |
| 27 | 6 | 0.645 |  | 30 | 14 | 0.633 |  | 39 | 13 | 0.622 |  | 25 | 21 | 0.612 |  | 21 | 13 | 0.602 |
| 19 | 6 | 0.644 |  | 31 | 26 | 0.632 |  | 21 | 4 | 0.622 |  | 36 | 19 | 0.612 |  | 35 | 24 | 0.602 |
| 8 | 2 | 0.644 |  | 26 | 1 | 0.631 |  | 19 | 13 | 0.622 |  | 30 | 3 | 0.612 |  | 8 | 6 | 0.602 |
| 40 | 32 | 0.644 |  | 21 | 17 | 0.631 |  | 40 | 35 | 0.622 |  | 26 | 13 | 0.612 |  | 39 | 21 | 0.601 |
| 10 | 7 | 0.644 |  | 12 | 3 | 0.631 |  | 39 | 26 | 0.622 |  | 7 | 1 | 0.611 |  | 35 | 31 | 0.601 |
| 31 | 9 | 0.643 |  | 9 | 5 | 0.630 |  | 39 | 22 | 0.621 |  | 19 | 3 | 0.610 |  | 21 | 9 | 0.601 |
| 26 | 11 | 0.643 |  | 21 | 14 | 0.630 |  | 15 | 10 | 0.620 |  | 35 | 13 | 0.610 |  | 39 | 3 | 0.601 |
| 27 | 20 | 0.643 |  | 16 | 7 | 0.630 |  | 40 | 17 | 0.619 |  | 28 | 16 | 0.610 |  | 40 | 23 | 0.601 |
| 32 | 6 | 0.642 |  | 27 | 3 | 0.630 |  | 39 | 35 | 0.619 |  | 35 | 9 | 0.610 |  | 28 | 12 | 0.601 |
| 18 | 9 | 0.642 |  | 35 | 1 | 0.629 |  | 17 | 12 | 0.619 |  | 17 | 6 | 0.610 |  | 17 | 1 | 0.601 |
| 39 | 14 | 0.642 |  | 36 | 15 | 0.629 |  | 33 | 4 | 0.618 |  | 40 | 6 | 0.610 |  | 40 | 29 | 0.600 |
| 34 | 5 | 0.642 |  | 35 | 16 | 0.629 |  | 38 | 17 | 0.618 |  | 7 | 5 | 0.609 |  | 40 | 25 | 0.600 |
| 38 | 8 | 0.642 |  | 37 | 28 | 0.628 |  | 37 | 29 | 0.618 |  | 38 | 27 | 0.609 |  | 32 | 12 | 0.600 |
| 12 | 7 | 0.642 |  | 27 | 12 | 0.628 |  | 27 | 1 | 0.618 |  | 33 | 6 | 0.609 |  | 30 | 12 | 0.600 |
| 40 | 21 | 0.642 |  | 12 | 6 | 0.628 |  | 40 | 31 | 0.618 |  | 37 | 30 | 0.608 |  | 27 | 25 | 0.600 |
| 21 | 12 | 0.642 |  | 18 | 17 | 0.628 |  | 34 | 23 | 0.617 |  | 34 | 17 | 0.608 |  | 22 | 7 | 0.600 |
| 15 | 9 | 0.642 |  | 31 | 18 | 0.627 |  | 34 | 16 | 0.617 |  | 29 | 11 | 0.608 |  | 30 | 13 | 0.599 |
| 30 | 17 | 0.642 |  | 28 | 11 | 0.627 |  | 18 | 2 | 0.617 |  | 19 | 17 | 0.608 |  | 30 | 5 | 0.599 |
| 33 | 10 | 0.642 |  | 12 | 1 | 0.627 |  | 14 | 12 | 0.617 |  | 14 | 6 | 0.608 |  | 25 | 20 | 0.599 |
| 31 | 25 | 0.642 |  | 39 | 33 | 0.627 |  | 29 | 6 | 0.617 |  | 21 | 3 | 0.608 |  | 40 | 24 | 0.599 |
| 30 | 15 | 0.641 |  | 33 | 14 | 0.627 |  | 29 | 23 | 0.617 |  | 20 | 1 | 0.608 |  | 16 | 9 | 0.599 |
| 29 | 15 | 0.641 |  | 36 | 7 | 0.626 |  | 40 | 19 | 0.617 |  | 17 | 9 | 0.608 |  | 9 | 4 | 0.599 |
| 23 | 20 | 0.641 |  | 37 | 10 | 0.625 |  | 34 | 1 | 0.617 |  | 14 | 1 | 0.608 |  | 40 | 5 | 0.599 |
| 13 | 12 | 0.641 |  | 33 | 5 | 0.625 |  | 39 | 17 | 0.617 |  | 29 | 14 | 0.607 |  | 20 | 12 | 0.599 |
| 33 | 22 | 0.640 |  | 32 | 16 | 0.625 |  | 30 | 1 | 0.617 |  | 26 | 18 | 0.607 |  | 33 | 1 | 0.599 |
| 22 | 12 | 0.640 |  | 30 | 23 | 0.625 |  | 20 | 16 | 0.617 |  | 39 | 12 | 0.607 |  | 36 | 16 | 0.598 |
| 36 | 2 | 0.639 |  | 30 | 16 | 0.625 |  | 31 | 1 | 0.616 |  | 27 | 10 | 0.607 |  | 35 | 18 | 0.598 |
| 36 | 17 | 0.639 |  | 12 | 5 | 0.625 |  | 28 | 19 | 0.616 |  | 15 | 3 | 0.607 |  | 25 | 9 | 0.598 |
| 40 | 12 | 0.639 |  | 11 | 5 | 0.625 |  | 40 | 13 | 0.616 |  | 38 | 31 | 0.607 |  | 32 | 19 | 0.597 |
| 23 | 21 | 0.639 |  | 24 | 12 | 0.624 |  | 23 | 9 | 0.616 |  | 4 | 2 | 0.607 |  | 29 | 19 | 0.597 |
| 39 | 37 | 0.638 |  | 33 | 13 | 0.624 |  | 39 | 32 | 0.615 |  | 38 | 9 | 0.606 |  | 7 | 2 | 0.597 |
| 38 | 33 | 0.637 |  | 30 | 19 | 0.624 |  | 31 | 20 | 0.615 |  | 26 | 12 | 0.605 |  | 26 | 25 | 0.596 |

| **Table S2-1:** The pairwise genetic distance coefficients estimated based on Jaccard distance coefficients based on **SCoT** data | | | | | | | | | | | | | | | | | | |
| --- | --- | --- | --- | --- | --- | --- | --- | --- | --- | --- | --- | --- | --- | --- | --- | --- | --- | --- |
| Genotypes | | distance |  | Genotypes | | distance |  | Genotypes | | distance |  | Genotypes | | distance |  | Genotypes | | distance |
| Id(i) | Id(j) | d(i,j) |  | Id(i) | Id(j) | d(i,j) |  | Id(i) | Id(j) | d(i,j) |  | Id(i) | Id(j) | d(i,j) |  | Id(i) | Id(j) | d(i,j) |
| 24 | 9 | 0.596 |  | 35 | 25 | 0.587 |  | 14 | 4 | 0.574 |  | 20 | 5 | 0.563 |  | 38 | 7 | 0.551 |
| 39 | 24 | 0.596 |  | 18 | 16 | 0.587 |  | 39 | 19 | 0.574 |  | 39 | 23 | 0.562 |  | 24 | 10 | 0.551 |
| 21 | 16 | 0.596 |  | 35 | 27 | 0.586 |  | 17 | 14 | 0.573 |  | 19 | 16 | 0.562 |  | 29 | 25 | 0.550 |
| 20 | 9 | 0.596 |  | 25 | 23 | 0.585 |  | 10 | 6 | 0.573 |  | 37 | 35 | 0.562 |  | 32 | 24 | 0.550 |
| 33 | 19 | 0.595 |  | 18 | 7 | 0.585 |  | 22 | 20 | 0.573 |  | 36 | 30 | 0.562 |  | 32 | 13 | 0.550 |
| 32 | 3 | 0.595 |  | 29 | 16 | 0.585 |  | 39 | 28 | 0.571 |  | 33 | 23 | 0.562 |  | 36 | 27 | 0.550 |
| 26 | 10 | 0.595 |  | 29 | 4 | 0.585 |  | 39 | 16 | 0.571 |  | 33 | 16 | 0.562 |  | 4 | 1 | 0.549 |
| 6 | 3 | 0.595 |  | 22 | 17 | 0.584 |  | 36 | 25 | 0.571 |  | 29 | 3 | 0.561 |  | 39 | 1 | 0.549 |
| 28 | 23 | 0.594 |  | 23 | 6 | 0.584 |  | 21 | 11 | 0.571 |  | 29 | 18 | 0.560 |  | 27 | 4 | 0.548 |
| 27 | 18 | 0.594 |  | 20 | 18 | 0.584 |  | 36 | 24 | 0.571 |  | 3 | 1 | 0.560 |  | 39 | 6 | 0.547 |
| 20 | 3 | 0.594 |  | 40 | 28 | 0.583 |  | 36 | 13 | 0.571 |  | 39 | 5 | 0.559 |  | 40 | 4 | 0.546 |
| 40 | 18 | 0.594 |  | 38 | 22 | 0.583 |  | 17 | 3 | 0.571 |  | 38 | 26 | 0.559 |  | 18 | 3 | 0.546 |
| 30 | 4 | 0.593 |  | 30 | 24 | 0.583 |  | 32 | 9 | 0.571 |  | 32 | 25 | 0.559 |  | 32 | 27 | 0.545 |
| 28 | 6 | 0.593 |  | 20 | 4 | 0.583 |  | 21 | 18 | 0.571 |  | 28 | 5 | 0.559 |  | 16 | 3 | 0.545 |
| 27 | 23 | 0.593 |  | 30 | 27 | 0.582 |  | 19 | 10 | 0.570 |  | 38 | 19 | 0.559 |  | 5 | 2 | 0.545 |
| 27 | 16 | 0.593 |  | 35 | 3 | 0.582 |  | 39 | 30 | 0.570 |  | 16 | 1 | 0.558 |  | 25 | 3 | 0.545 |
| 16 | 12 | 0.592 |  | 25 | 14 | 0.582 |  | 22 | 16 | 0.569 |  | 29 | 24 | 0.558 |  | 28 | 9 | 0.545 |
| 9 | 3 | 0.592 |  | 29 | 12 | 0.582 |  | 35 | 5 | 0.569 |  | 40 | 36 | 0.558 |  | 39 | 36 | 0.545 |
| 17 | 10 | 0.592 |  | 34 | 3 | 0.582 |  | 30 | 18 | 0.569 |  | 36 | 12 | 0.558 |  | 28 | 4 | 0.544 |
| 29 | 1 | 0.591 |  | 40 | 1 | 0.581 |  | 23 | 17 | 0.569 |  | 21 | 5 | 0.558 |  | 13 | 3 | 0.544 |
| 40 | 30 | 0.591 |  | 38 | 12 | 0.581 |  | 38 | 3 | 0.569 |  | 28 | 27 | 0.557 |  | 10 | 3 | 0.544 |
| 6 | 1 | 0.591 |  | 26 | 21 | 0.581 |  | 26 | 5 | 0.569 |  | 18 | 14 | 0.557 |  | 28 | 26 | 0.544 |
| 38 | 13 | 0.591 |  | 23 | 19 | 0.581 |  | 18 | 1 | 0.568 |  | 38 | 32 | 0.556 |  | 36 | 21 | 0.544 |
| 27 | 13 | 0.590 |  | 33 | 17 | 0.580 |  | 36 | 20 | 0.568 |  | 12 | 4 | 0.556 |  | 24 | 23 | 0.543 |
| 13 | 6 | 0.590 |  | 26 | 16 | 0.580 |  | 27 | 19 | 0.567 |  | 36 | 4 | 0.555 |  | 23 | 10 | 0.543 |
| 32 | 14 | 0.590 |  | 14 | 5 | 0.580 |  | 30 | 25 | 0.567 |  | 24 | 14 | 0.555 |  | 10 | 4 | 0.543 |
| 27 | 5 | 0.590 |  | 32 | 11 | 0.579 |  | 23 | 5 | 0.567 |  | 14 | 10 | 0.555 |  | 36 | 5 | 0.543 |
| 12 | 10 | 0.590 |  | 30 | 11 | 0.579 |  | 16 | 5 | 0.567 |  | 38 | 1 | 0.555 |  | 32 | 18 | 0.543 |
| 38 | 14 | 0.590 |  | 39 | 29 | 0.579 |  | 38 | 23 | 0.567 |  | 29 | 27 | 0.555 |  | 13 | 10 | 0.542 |
| 24 | 20 | 0.589 |  | 31 | 22 | 0.579 |  | 28 | 1 | 0.567 |  | 4 | 3 | 0.554 |  | 6 | 2 | 0.542 |
| 20 | 13 | 0.589 |  | 26 | 24 | 0.578 |  | 35 | 20 | 0.567 |  | 36 | 11 | 0.554 |  | 22 | 1 | 0.541 |
| 40 | 20 | 0.589 |  | 22 | 21 | 0.577 |  | 39 | 20 | 0.566 |  | 38 | 6 | 0.553 |  | 36 | 22 | 0.538 |
| 38 | 11 | 0.589 |  | 17 | 4 | 0.577 |  | 16 | 6 | 0.566 |  | 35 | 11 | 0.553 |  | 16 | 14 | 0.538 |
| 37 | 32 | 0.589 |  | 39 | 7 | 0.577 |  | 38 | 24 | 0.565 |  | 26 | 19 | 0.553 |  | 14 | 9 | 0.538 |
| 29 | 9 | 0.589 |  | 32 | 23 | 0.576 |  | 32 | 5 | 0.565 |  | 19 | 4 | 0.553 |  | 31 | 21 | 0.538 |
| 38 | 37 | 0.588 |  | 32 | 4 | 0.576 |  | 10 | 1 | 0.565 |  | 28 | 25 | 0.552 |  | 35 | 22 | 0.538 |
| 35 | 23 | 0.588 |  | 7 | 3 | 0.576 |  | 37 | 20 | 0.565 |  | 25 | 4 | 0.552 |  | 5 | 1 | 0.538 |
| 28 | 3 | 0.588 |  | 38 | 16 | 0.576 |  | 36 | 18 | 0.564 |  | 28 | 24 | 0.552 |  | 26 | 22 | 0.536 |
| 11 | 10 | 0.588 |  | 25 | 10 | 0.575 |  | 27 | 24 | 0.564 |  | 28 | 13 | 0.552 |  | 35 | 26 | 0.536 |
| 9 | 1 | 0.588 |  | 38 | 25 | 0.575 |  | 36 | 23 | 0.564 |  | 24 | 4 | 0.552 |  | 16 | 4 | 0.536 |
| 19 | 1 | 0.588 |  | 17 | 5 | 0.575 |  | 40 | 10 | 0.563 |  | 16 | 10 | 0.552 |  | 30 | 20 | 0.535 |
| 19 | 7 | 0.588 |  | 29 | 13 | 0.575 |  | 37 | 36 | 0.563 |  | 13 | 4 | 0.552 |  | 10 | 9 | 0.535 |
| 24 | 19 | 0.588 |  | 40 | 16 | 0.574 |  | 39 | 18 | 0.563 |  | 36 | 1 | 0.551 |  | 19 | 18 | 0.534 |
| 37 | 12 | 0.587 |  | 32 | 1 | 0.574 |  | 22 | 9 | 0.563 |  | 30 | 26 | 0.551 |  | 35 | 21 | 0.533 |

| **Table S2-1:** The pairwise genetic distance coefficients estimated based on Jaccard distance coefficients based on **SCoT** data | | | | | | | | | | | | | | |
| --- | --- | --- | --- | --- | --- | --- | --- | --- | --- | --- | --- | --- | --- | --- |
| Genotypes | | distance |  | Genotypes | | distance |  | Genotypes | | distance |  | Genotypes | | distance |
| Id(i) | Id(j) | d(i,j) |  | Id(i) | Id(j) | d(i,j) |  | Id(i) | Id(j) | d(i,j) |  | Id(i) | Id(j) | d(i,j) |
| 25 | 17 | 0.533 |  | 30 | 22 | 0.517 |  | 26 | 4 | 0.497 |  | 35 | 29 | 0.447 |
| 30 | 9 | 0.533 |  | 38 | 20 | 0.514 |  | 29 | 20 | 0.497 |  | 6 | 5 | 0.445 |
| 25 | 1 | 0.531 |  | 39 | 10 | 0.513 |  | 32 | 31 | 0.496 |  | 32 | 30 | 0.444 |
| 29 | 5 | 0.530 |  | 29 | 26 | 0.513 |  | 18 | 13 | 0.491 |  | 29 | 10 | 0.440 |
| 27 | 22 | 0.530 |  | 38 | 28 | 0.513 |  | 36 | 10 | 0.491 |  | 5 | 4 | 0.439 |
| 31 | 10 | 0.530 |  | 23 | 1 | 0.513 |  | 18 | 6 | 0.490 |  | 38 | 10 | 0.435 |
| 38 | 29 | 0.529 |  | 14 | 3 | 0.510 |  | 30 | 21 | 0.490 |  | 16 | 13 | 0.435 |
| 36 | 3 | 0.529 |  | 38 | 30 | 0.510 |  | 30 | 10 | 0.488 |  | 6 | 4 | 0.428 |
| 25 | 22 | 0.528 |  | 28 | 20 | 0.510 |  | 23 | 22 | 0.485 |  | 22 | 5 | 0.427 |
| 23 | 3 | 0.528 |  | 23 | 14 | 0.510 |  | 22 | 4 | 0.485 |  | 28 | 10 | 0.425 |
| 38 | 21 | 0.527 |  | 36 | 28 | 0.509 |  | 13 | 1 | 0.484 |  | 7 | 6 | 0.418 |
| 28 | 18 | 0.527 |  | 35 | 10 | 0.509 |  | 28 | 21 | 0.483 |  | 24 | 16 | 0.415 |
| 24 | 3 | 0.527 |  | 31 | 30 | 0.507 |  | 24 | 18 | 0.482 |  | 32 | 28 | 0.408 |
| 32 | 22 | 0.526 |  | 31 | 29 | 0.507 |  | 32 | 21 | 0.479 |  | 25 | 13 | 0.406 |
| 32 | 20 | 0.526 |  | 36 | 26 | 0.507 |  | 22 | 10 | 0.477 |  | 32 | 29 | 0.403 |
| 13 | 9 | 0.525 |  | 36 | 29 | 0.506 |  | 28 | 22 | 0.476 |  | 30 | 29 | 0.392 |
| 5 | 3 | 0.525 |  | 35 | 30 | 0.506 |  | 25 | 16 | 0.472 |  | 21 | 20 | 0.389 |
| 25 | 5 | 0.524 |  | 39 | 4 | 0.503 |  | 17 | 13 | 0.469 |  | 30 | 28 | 0.387 |
| 24 | 5 | 0.524 |  | 32 | 26 | 0.503 |  | 38 | 4 | 0.464 |  | 20 | 10 | 0.377 |
| 13 | 5 | 0.524 |  | 38 | 18 | 0.503 |  | 14 | 13 | 0.461 |  | 24 | 13 | 0.367 |
| 10 | 5 | 0.524 |  | 24 | 1 | 0.503 |  | 32 | 10 | 0.460 |  | 40 | 38 | 0.346 |
| 38 | 35 | 0.523 |  | 22 | 3 | 0.503 |  | 22 | 13 | 0.459 |  | 29 | 28 | 0.342 |
| 22 | 6 | 0.521 |  | 33 | 3 | 0.500 |  | 22 | 18 | 0.458 |  | 36 | 35 | 0.338 |
| 22 | 14 | 0.521 |  | 25 | 18 | 0.500 |  | 8 | 7 | 0.457 |  | 21 | 10 | 0.331 |
| 24 | 22 | 0.520 |  | 23 | 13 | 0.500 |  | 31 | 28 | 0.457 |  | 39 | 38 | 0.258 |
| 38 | 5 | 0.519 |  | 18 | 10 | 0.500 |  | 29 | 22 | 0.455 |  | 34 | 33 | 0.247 |
| 35 | 28 | 0.519 |  | 18 | 4 | 0.500 |  | 38 | 36 | 0.452 |  | 17 | 16 | 0.147 |
| 23 | 18 | 0.518 |  | 24 | 17 | 0.497 |  | 18 | 5 | 0.449 |  | 40 | 39 | 0.125 |
| 23 | 16 | 0.518 |  | 35 | 32 | 0.497 |  | 7 | 4 | 0.449 |  | 25 | 24 | 0.111 |
| 23 | 4 | 0.518 |  | 36 | 32 | 0.497 |  | 29 | 21 | 0.448 |  | 27 | 26 | 0.107 |

| **Table S2-2:** The pairwise genetic distance coefficients estimated based on Jaccard distance coefficients based on **URP** data | | | | | | | | | | | | | | | | | | |
| --- | --- | --- | --- | --- | --- | --- | --- | --- | --- | --- | --- | --- | --- | --- | --- | --- | --- | --- |
| Genotypes | | distance |  | Genotypes | | distance |  | Genotypes | | distance |  | Genotypes | | distance |  | Genotypes | | distance |
| Id(i) | Id(j) | d(i,j) |  | Id(i) | Id(j) | d(i,j) |  | Id(i) | Id(j) | d(i,j) |  | Id(i) | Id(j) | d(i,j) |  | Id(i) | Id(j) | d(i,j) |
| 16 | 9 | 0.872 |  | 40 | 27 | 0.800 |  | 37 | 27 | 0.776 |  | 22 | 15 | 0.740 |  | 31 | 15 | 0.719 |
| 39 | 9 | 0.865 |  | 26 | 3 | 0.800 |  | 33 | 27 | 0.774 |  | 40 | 12 | 0.740 |  | 40 | 6 | 0.719 |
| 40 | 9 | 0.862 |  | 25 | 9 | 0.799 |  | 21 | 7 | 0.773 |  | 37 | 7 | 0.739 |  | 40 | 13 | 0.718 |
| 38 | 9 | 0.862 |  | 9 | 6 | 0.799 |  | 27 | 3 | 0.773 |  | 29 | 25 | 0.739 |  | 29 | 28 | 0.718 |
| 27 | 9 | 0.861 |  | 34 | 26 | 0.799 |  | 30 | 26 | 0.772 |  | 30 | 29 | 0.739 |  | 36 | 15 | 0.717 |
| 36 | 9 | 0.860 |  | 15 | 7 | 0.799 |  | 27 | 13 | 0.771 |  | 35 | 15 | 0.738 |  | 35 | 19 | 0.717 |
| 26 | 9 | 0.860 |  | 26 | 12 | 0.798 |  | 26 | 16 | 0.770 |  | 40 | 15 | 0.738 |  | 29 | 11 | 0.716 |
| 26 | 25 | 0.858 |  | 26 | 21 | 0.797 |  | 17 | 15 | 0.770 |  | 18 | 7 | 0.738 |  | 29 | 18 | 0.716 |
| 11 | 9 | 0.855 |  | 26 | 15 | 0.797 |  | 31 | 27 | 0.768 |  | 32 | 12 | 0.737 |  | 35 | 2 | 0.716 |
| 9 | 1 | 0.852 |  | 20 | 9 | 0.797 |  | 32 | 26 | 0.766 |  | 32 | 15 | 0.736 |  | 28 | 12 | 0.716 |
| 18 | 9 | 0.849 |  | 13 | 9 | 0.796 |  | 25 | 15 | 0.764 |  | 40 | 28 | 0.735 |  | 29 | 19 | 0.715 |
| 15 | 9 | 0.849 |  | 26 | 13 | 0.795 |  | 23 | 15 | 0.763 |  | 34 | 15 | 0.735 |  | 14 | 7 | 0.715 |
| 26 | 1 | 0.848 |  | 33 | 26 | 0.792 |  | 39 | 26 | 0.762 |  | 30 | 15 | 0.735 |  | 38 | 7 | 0.715 |
| 30 | 9 | 0.847 |  | 27 | 19 | 0.792 |  | 37 | 26 | 0.761 |  | 18 | 15 | 0.734 |  | 29 | 22 | 0.714 |
| 17 | 9 | 0.840 |  | 34 | 9 | 0.791 |  | 29 | 14 | 0.760 |  | 15 | 8 | 0.734 |  | 29 | 17 | 0.714 |
| 27 | 25 | 0.838 |  | 26 | 6 | 0.791 |  | 38 | 27 | 0.759 |  | 37 | 25 | 0.734 |  | 37 | 34 | 0.714 |
| 14 | 9 | 0.833 |  | 31 | 26 | 0.790 |  | 25 | 12 | 0.759 |  | 39 | 25 | 0.733 |  | 12 | 10 | 0.714 |
| 35 | 26 | 0.831 |  | 29 | 27 | 0.790 |  | 40 | 25 | 0.757 |  | 27 | 20 | 0.733 |  | 36 | 7 | 0.713 |
| 27 | 12 | 0.830 |  | 9 | 7 | 0.790 |  | 26 | 17 | 0.757 |  | 20 | 7 | 0.732 |  | 21 | 5 | 0.713 |
| 27 | 15 | 0.828 |  | 24 | 9 | 0.789 |  | 28 | 26 | 0.756 |  | 28 | 27 | 0.732 |  | 15 | 10 | 0.712 |
| 37 | 9 | 0.827 |  | 26 | 18 | 0.788 |  | 22 | 12 | 0.756 |  | 27 | 11 | 0.732 |  | 19 | 15 | 0.712 |
| 12 | 9 | 0.825 |  | 29 | 15 | 0.787 |  | 26 | 4 | 0.755 |  | 19 | 7 | 0.732 |  | 36 | 27 | 0.711 |
| 10 | 9 | 0.823 |  | 26 | 19 | 0.787 |  | 17 | 7 | 0.751 |  | 15 | 4 | 0.732 |  | 40 | 24 | 0.711 |
| 9 | 3 | 0.822 |  | 27 | 6 | 0.787 |  | 26 | 11 | 0.750 |  | 11 | 7 | 0.731 |  | 32 | 7 | 0.711 |
| 9 | 2 | 0.821 |  | 22 | 9 | 0.787 |  | 29 | 13 | 0.749 |  | 30 | 7 | 0.731 |  | 24 | 15 | 0.710 |
| 27 | 1 | 0.821 |  | 39 | 27 | 0.787 |  | 16 | 7 | 0.749 |  | 15 | 13 | 0.731 |  | 6 | 2 | 0.710 |
| 31 | 9 | 0.821 |  | 27 | 21 | 0.787 |  | 15 | 14 | 0.749 |  | 19 | 12 | 0.730 |  | 12 | 4 | 0.710 |
| 27 | 7 | 0.820 |  | 28 | 9 | 0.787 |  | 32 | 27 | 0.748 |  | 29 | 10 | 0.729 |  | 39 | 13 | 0.709 |
| 35 | 27 | 0.819 |  | 27 | 14 | 0.786 |  | 25 | 19 | 0.747 |  | 38 | 29 | 0.729 |  | 20 | 12 | 0.709 |
| 26 | 8 | 0.819 |  | 15 | 3 | 0.786 |  | 30 | 27 | 0.747 |  | 18 | 12 | 0.729 |  | 8 | 7 | 0.709 |
| 21 | 9 | 0.819 |  | 40 | 26 | 0.786 |  | 27 | 17 | 0.747 |  | 35 | 5 | 0.729 |  | 28 | 15 | 0.707 |
| 26 | 22 | 0.816 |  | 29 | 9 | 0.786 |  | 36 | 26 | 0.747 |  | 31 | 7 | 0.728 |  | 29 | 3 | 0.707 |
| 27 | 8 | 0.814 |  | 26 | 5 | 0.786 |  | 15 | 1 | 0.746 |  | 35 | 7 | 0.727 |  | 34 | 12 | 0.707 |
| 33 | 9 | 0.814 |  | 27 | 5 | 0.782 |  | 26 | 10 | 0.745 |  | 29 | 21 | 0.725 |  | 40 | 29 | 0.706 |
| 26 | 14 | 0.813 |  | 34 | 27 | 0.780 |  | 28 | 7 | 0.745 |  | 33 | 15 | 0.724 |  | 12 | 8 | 0.706 |
| 9 | 4 | 0.813 |  | 26 | 23 | 0.780 |  | 27 | 10 | 0.744 |  | 7 | 2 | 0.723 |  | 29 | 2 | 0.705 |
| 9 | 8 | 0.811 |  | 26 | 2 | 0.779 |  | 15 | 2 | 0.744 |  | 16 | 15 | 0.723 |  | 19 | 13 | 0.705 |
| 35 | 9 | 0.808 |  | 27 | 24 | 0.778 |  | 35 | 29 | 0.743 |  | 10 | 7 | 0.723 |  | 19 | 2 | 0.704 |
| 26 | 7 | 0.808 |  | 27 | 4 | 0.778 |  | 12 | 6 | 0.743 |  | 7 | 6 | 0.722 |  | 38 | 15 | 0.704 |
| 23 | 9 | 0.808 |  | 27 | 18 | 0.777 |  | 26 | 20 | 0.742 |  | 29 | 5 | 0.722 |  | 19 | 5 | 0.703 |
| 19 | 9 | 0.804 |  | 27 | 2 | 0.776 |  | 36 | 12 | 0.741 |  | 29 | 24 | 0.721 |  | 37 | 15 | 0.703 |
| 32 | 9 | 0.803 |  | 38 | 26 | 0.776 |  | 27 | 23 | 0.741 |  | 36 | 29 | 0.720 |  | 15 | 12 | 0.703 |
| 9 | 5 | 0.803 |  | 27 | 22 | 0.776 |  | 29 | 26 | 0.741 |  | 39 | 12 | 0.719 |  | 32 | 17 | 0.701 |
| 26 | 24 | 0.802 |  | 27 | 16 | 0.776 |  | 15 | 6 | 0.741 |  | 24 | 12 | 0.719 |  | 29 | 7 | 0.701 |

| **Table S2-2:** The pairwise genetic distance coefficients estimated based on Jaccard distance coefficients based on **URP** data | | | | | | | | | | | | | | | | | | |
| --- | --- | --- | --- | --- | --- | --- | --- | --- | --- | --- | --- | --- | --- | --- | --- | --- | --- | --- |
| Genotypes | | distance |  | Genotypes | | distance |  | Genotypes | | distance |  | Genotypes | | distance |  | Genotypes | | distance |
| Id(i) | Id(j) | d(i,j) |  | Id(i) | Id(j) | d(i,j) |  | Id(i) | Id(j) | d(i,j) |  | Id(i) | Id(j) | d(i,j) |  | Id(i) | Id(j) | d(i,j) |
| 22 | 7 | 0.701 |  | 32 | 2 | 0.686 |  | 39 | 2 | 0.675 |  | 10 | 5 | 0.665 |  | 39 | 17 | 0.655 |
| 36 | 1 | 0.700 |  | 6 | 1 | 0.686 |  | 40 | 22 | 0.674 |  | 35 | 1 | 0.665 |  | 25 | 21 | 0.654 |
| 21 | 12 | 0.700 |  | 40 | 35 | 0.686 |  | 23 | 12 | 0.674 |  | 37 | 1 | 0.665 |  | 22 | 1 | 0.654 |
| 25 | 17 | 0.699 |  | 40 | 2 | 0.686 |  | 34 | 25 | 0.674 |  | 39 | 22 | 0.665 |  | 20 | 3 | 0.654 |
| 12 | 7 | 0.699 |  | 29 | 4 | 0.686 |  | 33 | 29 | 0.673 |  | 6 | 5 | 0.665 |  | 34 | 31 | 0.654 |
| 39 | 28 | 0.699 |  | 30 | 2 | 0.686 |  | 14 | 12 | 0.673 |  | 39 | 34 | 0.665 |  | 36 | 17 | 0.654 |
| 40 | 7 | 0.699 |  | 7 | 3 | 0.685 |  | 7 | 4 | 0.673 |  | 37 | 2 | 0.665 |  | 37 | 3 | 0.653 |
| 35 | 25 | 0.697 |  | 23 | 7 | 0.685 |  | 21 | 19 | 0.672 |  | 40 | 3 | 0.665 |  | 25 | 8 | 0.653 |
| 29 | 23 | 0.697 |  | 15 | 5 | 0.684 |  | 20 | 5 | 0.672 |  | 15 | 11 | 0.665 |  | 18 | 3 | 0.653 |
| 29 | 6 | 0.697 |  | 35 | 34 | 0.684 |  | 35 | 16 | 0.672 |  | 10 | 8 | 0.663 |  | 24 | 20 | 0.652 |
| 36 | 14 | 0.696 |  | 37 | 17 | 0.683 |  | 30 | 5 | 0.672 |  | 38 | 5 | 0.663 |  | 35 | 13 | 0.652 |
| 28 | 19 | 0.695 |  | 19 | 17 | 0.683 |  | 21 | 6 | 0.672 |  | 36 | 25 | 0.663 |  | 37 | 6 | 0.651 |
| 12 | 2 | 0.695 |  | 13 | 12 | 0.683 |  | 14 | 11 | 0.672 |  | 40 | 37 | 0.662 |  | 36 | 18 | 0.651 |
| 39 | 24 | 0.695 |  | 29 | 8 | 0.683 |  | 37 | 13 | 0.672 |  | 31 | 5 | 0.662 |  | 31 | 6 | 0.651 |
| 12 | 5 | 0.695 |  | 34 | 30 | 0.683 |  | 24 | 19 | 0.672 |  | 38 | 25 | 0.662 |  | 31 | 19 | 0.650 |
| 31 | 29 | 0.695 |  | 21 | 15 | 0.683 |  | 35 | 18 | 0.672 |  | 21 | 8 | 0.662 |  | 25 | 3 | 0.650 |
| 19 | 1 | 0.694 |  | 25 | 16 | 0.683 |  | 37 | 29 | 0.671 |  | 35 | 17 | 0.661 |  | 34 | 10 | 0.650 |
| 30 | 19 | 0.694 |  | 22 | 10 | 0.682 |  | 35 | 12 | 0.671 |  | 11 | 1 | 0.661 |  | 28 | 3 | 0.650 |
| 19 | 10 | 0.693 |  | 34 | 21 | 0.682 |  | 39 | 14 | 0.671 |  | 14 | 6 | 0.661 |  | 36 | 22 | 0.649 |
| 16 | 12 | 0.693 |  | 20 | 17 | 0.682 |  | 30 | 12 | 0.671 |  | 39 | 30 | 0.661 |  | 33 | 10 | 0.649 |
| 11 | 3 | 0.693 |  | 12 | 3 | 0.681 |  | 19 | 11 | 0.670 |  | 25 | 5 | 0.661 |  | 24 | 2 | 0.649 |
| 40 | 30 | 0.693 |  | 31 | 2 | 0.681 |  | 22 | 6 | 0.670 |  | 39 | 32 | 0.661 |  | 39 | 8 | 0.649 |
| 17 | 11 | 0.693 |  | 36 | 6 | 0.681 |  | 21 | 1 | 0.670 |  | 39 | 36 | 0.661 |  | 38 | 2 | 0.649 |
| 34 | 19 | 0.692 |  | 35 | 6 | 0.681 |  | 34 | 32 | 0.670 |  | 28 | 18 | 0.660 |  | 36 | 34 | 0.649 |
| 37 | 24 | 0.692 |  | 40 | 17 | 0.681 |  | 20 | 8 | 0.670 |  | 30 | 6 | 0.660 |  | 14 | 10 | 0.649 |
| 32 | 1 | 0.692 |  | 7 | 1 | 0.681 |  | 25 | 10 | 0.670 |  | 21 | 17 | 0.660 |  | 37 | 22 | 0.648 |
| 37 | 8 | 0.692 |  | 22 | 8 | 0.680 |  | 39 | 7 | 0.669 |  | 14 | 5 | 0.659 |  | 34 | 6 | 0.648 |
| 39 | 35 | 0.692 |  | 20 | 2 | 0.679 |  | 12 | 1 | 0.669 |  | 40 | 34 | 0.659 |  | 33 | 20 | 0.648 |
| 20 | 15 | 0.692 |  | 32 | 5 | 0.679 |  | 11 | 5 | 0.669 |  | 30 | 18 | 0.659 |  | 32 | 14 | 0.648 |
| 34 | 29 | 0.692 |  | 40 | 36 | 0.679 |  | 22 | 2 | 0.669 |  | 10 | 2 | 0.658 |  | 20 | 14 | 0.648 |
| 25 | 2 | 0.691 |  | 29 | 20 | 0.679 |  | 11 | 4 | 0.668 |  | 37 | 18 | 0.658 |  | 40 | 32 | 0.647 |
| 19 | 14 | 0.691 |  | 34 | 7 | 0.679 |  | 25 | 18 | 0.668 |  | 24 | 7 | 0.657 |  | 40 | 20 | 0.647 |
| 39 | 15 | 0.690 |  | 32 | 16 | 0.679 |  | 38 | 34 | 0.668 |  | 32 | 23 | 0.657 |  | 6 | 3 | 0.646 |
| 22 | 5 | 0.690 |  | 25 | 7 | 0.679 |  | 40 | 31 | 0.667 |  | 19 | 16 | 0.657 |  | 39 | 3 | 0.646 |
| 25 | 1 | 0.689 |  | 20 | 16 | 0.679 |  | 37 | 36 | 0.667 |  | 36 | 23 | 0.657 |  | 32 | 29 | 0.646 |
| 17 | 12 | 0.689 |  | 13 | 6 | 0.678 |  | 37 | 35 | 0.667 |  | 30 | 8 | 0.657 |  | 34 | 5 | 0.646 |
| 36 | 19 | 0.689 |  | 29 | 16 | 0.677 |  | 37 | 19 | 0.667 |  | 40 | 14 | 0.656 |  | 32 | 6 | 0.646 |
| 37 | 5 | 0.688 |  | 29 | 1 | 0.677 |  | 31 | 12 | 0.667 |  | 35 | 8 | 0.656 |  | 21 | 14 | 0.646 |
| 33 | 12 | 0.688 |  | 25 | 6 | 0.677 |  | 29 | 12 | 0.667 |  | 21 | 16 | 0.656 |  | 11 | 8 | 0.646 |
| 28 | 6 | 0.688 |  | 39 | 6 | 0.677 |  | 21 | 3 | 0.667 |  | 22 | 19 | 0.656 |  | 19 | 3 | 0.645 |
| 20 | 19 | 0.688 |  | 25 | 20 | 0.677 |  | 12 | 11 | 0.667 |  | 11 | 6 | 0.656 |  | 35 | 24 | 0.645 |
| 28 | 5 | 0.687 |  | 11 | 2 | 0.676 |  | 28 | 8 | 0.665 |  | 18 | 2 | 0.656 |  | 25 | 11 | 0.645 |
| 37 | 16 | 0.687 |  | 37 | 33 | 0.676 |  | 30 | 4 | 0.665 |  | 34 | 13 | 0.655 |  | 36 | 24 | 0.645 |
| 36 | 5 | 0.687 |  | 16 | 11 | 0.676 |  | 22 | 17 | 0.665 |  | 30 | 3 | 0.655 |  | 36 | 13 | 0.645 |

| **Table S2-2:** The pairwise genetic distance coefficients estimated based on Jaccard distance coefficients based on **URP** data | | | | | | | | | | | | | | | | | | |
| --- | --- | --- | --- | --- | --- | --- | --- | --- | --- | --- | --- | --- | --- | --- | --- | --- | --- | --- |
| Genotypes | | distance |  | Genotypes | | distance |  | Genotypes | | distance |  | Genotypes | | distance |  | Genotypes | | distance |
| Id(i) | Id(j) | d(i,j) |  | Id(i) | Id(j) | d(i,j) |  | Id(i) | Id(j) | d(i,j) |  | Id(i) | Id(j) | d(i,j) |  | Id(i) | Id(j) | d(i,j) |
| 34 | 28 | 0.645 |  | 31 | 8 | 0.636 |  | 32 | 8 | 0.629 |  | 16 | 5 | 0.615 |  | 24 | 21 | 0.603 |
| 40 | 19 | 0.645 |  | 23 | 6 | 0.636 |  | 38 | 4 | 0.628 |  | 37 | 32 | 0.615 |  | 32 | 28 | 0.602 |
| 24 | 6 | 0.645 |  | 25 | 14 | 0.636 |  | 24 | 11 | 0.628 |  | 16 | 14 | 0.615 |  | 24 | 10 | 0.602 |
| 40 | 23 | 0.644 |  | 23 | 21 | 0.635 |  | 23 | 19 | 0.628 |  | 39 | 33 | 0.614 |  | 40 | 10 | 0.601 |
| 39 | 29 | 0.644 |  | 34 | 20 | 0.635 |  | 10 | 3 | 0.628 |  | 17 | 10 | 0.614 |  | 24 | 1 | 0.601 |
| 17 | 5 | 0.644 |  | 32 | 25 | 0.635 |  | 36 | 8 | 0.627 |  | 18 | 6 | 0.614 |  | 13 | 1 | 0.601 |
| 20 | 4 | 0.644 |  | 35 | 4 | 0.635 |  | 32 | 30 | 0.627 |  | 39 | 5 | 0.613 |  | 31 | 17 | 0.601 |
| 30 | 22 | 0.644 |  | 14 | 2 | 0.635 |  | 32 | 13 | 0.626 |  | 39 | 20 | 0.613 |  | 40 | 1 | 0.600 |
| 40 | 4 | 0.643 |  | 40 | 16 | 0.635 |  | 23 | 10 | 0.626 |  | 38 | 33 | 0.613 |  | 35 | 21 | 0.600 |
| 28 | 14 | 0.643 |  | 38 | 8 | 0.635 |  | 28 | 22 | 0.626 |  | 28 | 21 | 0.613 |  | 31 | 18 | 0.600 |
| 36 | 16 | 0.643 |  | 33 | 30 | 0.635 |  | 33 | 6 | 0.625 |  | 37 | 23 | 0.613 |  | 38 | 24 | 0.599 |
| 31 | 4 | 0.643 |  | 16 | 6 | 0.635 |  | 25 | 22 | 0.624 |  | 32 | 24 | 0.613 |  | 36 | 28 | 0.599 |
| 35 | 22 | 0.642 |  | 13 | 2 | 0.634 |  | 40 | 33 | 0.624 |  | 22 | 21 | 0.613 |  | 37 | 20 | 0.599 |
| 39 | 23 | 0.642 |  | 40 | 8 | 0.634 |  | 20 | 6 | 0.624 |  | 40 | 18 | 0.612 |  | 34 | 23 | 0.598 |
| 40 | 5 | 0.642 |  | 30 | 17 | 0.634 |  | 36 | 3 | 0.624 |  | 10 | 6 | 0.611 |  | 18 | 1 | 0.598 |
| 24 | 5 | 0.642 |  | 30 | 14 | 0.634 |  | 25 | 4 | 0.624 |  | 21 | 4 | 0.611 |  | 35 | 30 | 0.597 |
| 13 | 11 | 0.642 |  | 39 | 37 | 0.634 |  | 17 | 6 | 0.624 |  | 24 | 18 | 0.611 |  | 4 | 2 | 0.596 |
| 33 | 32 | 0.641 |  | 17 | 14 | 0.634 |  | 37 | 30 | 0.623 |  | 39 | 18 | 0.611 |  | 28 | 11 | 0.596 |
| 36 | 2 | 0.641 |  | 31 | 14 | 0.634 |  | 8 | 5 | 0.623 |  | 19 | 6 | 0.611 |  | 13 | 10 | 0.595 |
| 33 | 31 | 0.641 |  | 18 | 11 | 0.634 |  | 22 | 20 | 0.622 |  | 31 | 1 | 0.611 |  | 32 | 21 | 0.595 |
| 35 | 33 | 0.641 |  | 20 | 13 | 0.633 |  | 38 | 22 | 0.622 |  | 14 | 3 | 0.610 |  | 35 | 14 | 0.594 |
| 34 | 18 | 0.641 |  | 38 | 17 | 0.633 |  | 38 | 19 | 0.622 |  | 33 | 28 | 0.610 |  | 8 | 1 | 0.594 |
| 37 | 28 | 0.640 |  | 28 | 1 | 0.633 |  | 14 | 8 | 0.622 |  | 3 | 2 | 0.610 |  | 32 | 10 | 0.594 |
| 36 | 33 | 0.640 |  | 23 | 2 | 0.632 |  | 33 | 5 | 0.621 |  | 18 | 17 | 0.610 |  | 40 | 21 | 0.594 |
| 32 | 3 | 0.640 |  | 22 | 4 | 0.632 |  | 34 | 2 | 0.621 |  | 34 | 8 | 0.609 |  | 35 | 28 | 0.593 |
| 23 | 11 | 0.640 |  | 38 | 3 | 0.632 |  | 22 | 18 | 0.621 |  | 37 | 4 | 0.608 |  | 21 | 18 | 0.593 |
| 31 | 3 | 0.640 |  | 36 | 30 | 0.632 |  | 36 | 4 | 0.619 |  | 28 | 17 | 0.608 |  | 31 | 21 | 0.592 |
| 38 | 14 | 0.640 |  | 28 | 25 | 0.631 |  | 13 | 7 | 0.619 |  | 21 | 2 | 0.608 |  | 17 | 8 | 0.591 |
| 33 | 21 | 0.639 |  | 16 | 10 | 0.631 |  | 5 | 3 | 0.619 |  | 13 | 8 | 0.608 |  | 38 | 32 | 0.591 |
| 22 | 11 | 0.639 |  | 10 | 1 | 0.631 |  | 33 | 18 | 0.619 |  | 39 | 16 | 0.608 |  | 13 | 5 | 0.590 |
| 18 | 14 | 0.639 |  | 33 | 19 | 0.631 |  | 28 | 16 | 0.619 |  | 22 | 14 | 0.608 |  | 39 | 1 | 0.590 |
| 37 | 14 | 0.638 |  | 18 | 13 | 0.631 |  | 6 | 4 | 0.618 |  | 30 | 25 | 0.607 |  | 3 | 1 | 0.590 |
| 34 | 11 | 0.638 |  | 5 | 4 | 0.631 |  | 22 | 16 | 0.618 |  | 34 | 24 | 0.607 |  | 40 | 38 | 0.589 |
| 28 | 2 | 0.638 |  | 2 | 1 | 0.631 |  | 18 | 5 | 0.618 |  | 5 | 1 | 0.607 |  | 18 | 4 | 0.589 |
| 23 | 20 | 0.638 |  | 10 | 4 | 0.631 |  | 35 | 32 | 0.618 |  | 39 | 19 | 0.607 |  | 37 | 10 | 0.587 |
| 5 | 2 | 0.638 |  | 21 | 13 | 0.631 |  | 28 | 4 | 0.618 |  | 39 | 11 | 0.606 |  | 35 | 31 | 0.587 |
| 38 | 12 | 0.637 |  | 39 | 31 | 0.630 |  | 34 | 22 | 0.617 |  | 36 | 31 | 0.606 |  | 30 | 1 | 0.586 |
| 32 | 19 | 0.637 |  | 30 | 16 | 0.630 |  | 38 | 18 | 0.617 |  | 34 | 14 | 0.606 |  | 38 | 13 | 0.585 |
| 38 | 6 | 0.637 |  | 8 | 2 | 0.630 |  | 19 | 4 | 0.617 |  | 8 | 3 | 0.606 |  | 34 | 1 | 0.585 |
| 33 | 7 | 0.637 |  | 33 | 25 | 0.630 |  | 35 | 3 | 0.617 |  | 33 | 2 | 0.605 |  | 8 | 4 | 0.585 |
| 33 | 11 | 0.637 |  | 7 | 5 | 0.629 |  | 37 | 12 | 0.616 |  | 31 | 16 | 0.604 |  | 13 | 4 | 0.585 |
| 20 | 1 | 0.637 |  | 32 | 4 | 0.629 |  | 40 | 11 | 0.616 |  | 32 | 18 | 0.603 |  | 18 | 10 | 0.585 |
| 32 | 22 | 0.636 |  | 38 | 16 | 0.629 |  | 35 | 23 | 0.616 |  | 30 | 21 | 0.603 |  | 31 | 22 | 0.585 |
| 22 | 3 | 0.636 |  | 38 | 1 | 0.629 |  | 39 | 4 | 0.615 |  | 20 | 18 | 0.603 |  | 19 | 8 | 0.585 |

| **Table S2-2:** The pairwise genetic distance coefficients estimated based on Jaccard distance coefficients based on **URP** data | | | | | | | | | | | | | | | |
| --- | --- | --- | --- | --- | --- | --- | --- | --- | --- | --- | --- | --- | --- | --- | --- |
| Genotypes | | distance |  | Genotypes | | distance |  | Genotypes | | distance |  | Genotypes | | distance |  |
| Id(i) | Id(j) | d(i,j) |  | Id(i) | Id(j) | d(i,j) |  | Id(i) | Id(j) | d(i,j) |  | Id(i) | Id(j) | d(i,j) |  |
| 4 | 1 | 0.585 |  | 34 | 4 | 0.567 |  | 18 | 8 | 0.544 |  | 38 | 21 | 0.503 |  |
| 39 | 10 | 0.584 |  | 24 | 3 | 0.567 |  | 33 | 14 | 0.542 |  | 33 | 24 | 0.503 |  |
| 32 | 11 | 0.584 |  | 18 | 16 | 0.567 |  | 16 | 4 | 0.542 |  | 34 | 16 | 0.500 |  |
| 25 | 23 | 0.583 |  | 17 | 1 | 0.566 |  | 31 | 23 | 0.542 |  | 38 | 36 | 0.492 |  |
| 17 | 13 | 0.583 |  | 28 | 20 | 0.566 |  | 19 | 18 | 0.538 |  | 33 | 4 | 0.491 |  |
| 31 | 25 | 0.583 |  | 24 | 8 | 0.565 |  | 23 | 16 | 0.538 |  | 31 | 28 | 0.490 |  |
| 28 | 13 | 0.583 |  | 17 | 4 | 0.565 |  | 30 | 24 | 0.536 |  | 21 | 11 | 0.488 |  |
| 37 | 31 | 0.582 |  | 23 | 3 | 0.565 |  | 17 | 3 | 0.536 |  | 38 | 31 | 0.485 |  |
| 14 | 4 | 0.581 |  | 36 | 32 | 0.564 |  | 33 | 17 | 0.509 |  | 21 | 20 | 0.483 |  |
| 23 | 17 | 0.581 |  | 39 | 38 | 0.563 |  | 31 | 10 | 0.505 |  | 31 | 11 | 0.479 |  |
| 35 | 11 | 0.581 |  | 30 | 20 | 0.563 |  | 38 | 35 | 0.535 |  | 20 | 11 | 0.477 |  |
| 35 | 20 | 0.581 |  | 37 | 21 | 0.562 |  | 24 | 16 | 0.533 |  | 30 | 28 | 0.476 |  |
| 33 | 22 | 0.580 |  | 28 | 24 | 0.562 |  | 36 | 20 | 0.531 |  | 24 | 23 | 0.474 |  |
| 23 | 22 | 0.579 |  | 36 | 10 | 0.561 |  | 24 | 4 | 0.530 |  | 11 | 10 | 0.469 |  |
| 38 | 23 | 0.576 |  | 34 | 4 | 0.567 |  | 36 | 11 | 0.529 |  | 38 | 10 | 0.460 |  |
| 36 | 35 | 0.576 |  | 38 | 28 | 0.560 |  | 16 | 8 | 0.529 |  | 23 | 14 | 0.460 |  |
| 36 | 21 | 0.575 |  | 30 | 10 | 0.560 |  | 28 | 10 | 0.528 |  | 21 | 10 | 0.459 |  |
| 39 | 21 | 0.575 |  | 28 | 23 | 0.559 |  | 16 | 1 | 0.527 |  | 23 | 13 | 0.458 |  |
| 37 | 11 | 0.575 |  | 24 | 22 | 0.559 |  | 31 | 20 | 0.527 |  | 20 | 10 | 0.457 |  |
| 13 | 3 | 0.574 |  | 23 | 18 | 0.559 |  | 38 | 20 | 0.524 |  | 31 | 30 | 0.447 |  |
| 34 | 17 | 0.574 |  | 30 | 13 | 0.558 |  | 30 | 11 | 0.522 |  | 33 | 16 | 0.429 |  |
| 22 | 13 | 0.574 |  | 31 | 24 | 0.558 |  | 14 | 13 | 0.522 |  | 25 | 13 | 0.422 |  |
| 23 | 5 | 0.572 |  | 33 | 13 | 0.557 |  | 16 | 2 | 0.516 |  | 38 | 11 | 0.399 |  |
| 8 | 6 | 0.571 |  | 16 | 13 | 0.556 |  | 33 | 23 | 0.516 |  | 33 | 3 | 0.382 |  |
| 33 | 8 | 0.571 |  | 23 | 4 | 0.552 |  | 14 | 1 | 0.515 |  | 25 | 24 | 0.294 |  |
| 30 | 23 | 0.570 |  | 31 | 13 | 0.551 |  | 23 | 1 | 0.514 |  | 24 | 13 | 0.250 |  |
| 35 | 10 | 0.570 |  | 17 | 2 | 0.550 |  | 33 | 1 | 0.512 |  | 27 | 26 | 0.236 |  |
| 32 | 20 | 0.568 |  | 32 | 31 | 0.548 |  | 38 | 30 | 0.511 |  | 17 | 16 | 0.190 |  |
| 24 | 17 | 0.568 |  | 23 | 8 | 0.547 |  | 16 | 3 | 0.503 |  | 34 | 33 | 0.189 |  |
| 24 | 14 | 0.568 |  | 4 | 3 | 0.545 |  | 34 | 3 | 0.503 |  | 40 | 39 | 0.181 |  |
