# Supplementary material for: Genetic diversity analysis in a mini core collection of Damask rose (Rosa damascena Mill.) germplasm from Iran using URP and SCoT markers
Source: J Genet Eng Biotechnol. 2021 Sep 30;19:144. doi: 10.1186/s43141-021-00247-7 (PMC8484433; doi:10.1186/s43141-021-00247-7)
Supplement: Supplementary file 3 — Additional file 3: Supplementary figure1. Mantel correlation. The correlation between SCoT and URP data for all 40 accessions across five regions [file 43141_2021_247_MOESM3_ESM.docx]

The correlation between SCoT and URP data for all 40 accessions across five regions
